# CYB5R3 functions as a tumor suppressor by inducing ER stress-mediated apoptosis in lung cancer cells via the PERK-ATF4 and IRE1α-JNK pathways

**DOI:** 10.1038/s12276-024-01155-9

**Published:** 2024-01-22

**Authors:** Joo-Young Im, Soo Jin Kim, Jong-Lyul Park, Tae-Hee Han, Woo-il Kim, Inhyub Kim, Bomin Ko, So-Young Chun, Mi-Jung Kang, Bo-Kyung Kim, Sol A. Jeon, Seon-Kyu Kim, Incheol Ryu, Seon-Young Kim, Ki-Hoan Nam, Inah Hwang, Hyun Seung Ban, Misun Won

**Affiliations:** 1 Personalized Genomic Medicine Research Center, Korea Research Institute of Bioscience and Biotechnology (KRIBB), Daejeon, 34141 Republic of Korea; 2 Chungnam National University Sejong Hospital (CNUSH), Sejong, 30099 Republic of Korea; 3 Aging Convergence Research Center, KRIBB, Daejeon, 34141 Republic of Korea; 4 Biotherapeutics Translational Research Center, KRIBB, Daejeon, 34141 Republic of Korea; 5 KRIBB School of Bioscience, Korea University of Science and Technology, Daejeon, 34113 Republic of Korea; 6 R&D Center, OneCureGEN Co., Ltd., Daejeon, 34141 Republic of Korea; 7 YD Global Life Science Co., Ltd., Seongnam-si, Gyeonggi-do 13207 Republic of Korea; 8 Laboratory Animal Resource & Research Center, KRIBB, Cheongju, Chungbuk Republic of Korea; 9 Graduate School of Pharmaceutical Sciences, College of Pharmacy, Ewha Womans University, Seoul, 03760 Republic of Korea

**Keywords:** Tumour biomarkers, PolyADP-ribosylation, Metabolomics, Cellular signalling networks

## Abstract

Cytochrome b5 reductase 3 (CYB5R3) is involved in various cellular metabolic processes, including fatty acid synthesis and drug metabolism. However, the role of CYB5R3 in cancer development remains poorly understood. Here, we show that CYB5R3 expression is downregulated in human lung cancer cell lines and tissues. Adenoviral overexpression of CYB5R3 suppresses lung cancer cell growth in vitro and in vivo. However, CYB5R3 deficiency promotes tumorigenesis and metastasis in mouse models. Transcriptome analysis revealed that apoptosis- and endoplasmic reticulum (ER) stress-related genes are upregulated in CYB5R3-overexpressing lung cancer cells. Metabolomic analysis revealed that CYB5R3 overexpression increased the production of nicotinamide adenine dinucleotide (NAD^+^) and oxidized glutathione (GSSG). Ectopic CYB5R3 is mainly localized in the ER, where CYB5R3-dependent ER stress signaling is induced via activation of protein kinase RNA-like ER kinase (PERK) and inositol-requiring enzyme 1 alpha (IRE1α). Moreover, NAD^+^ activates poly (ADP-ribose) polymerase16 (PARP16), an ER-resident protein, to promote ADP-ribosylation of PERK and IRE1α and induce ER stress. In addition, CYB5R3 induces the generation of reactive oxygen species and caspase-9-dependent intrinsic cell death. Our findings highlight the importance of CYB5R3 as a tumor suppressor for the development of CYB5R3-based therapeutics for lung cancer.

## The Role of CYB5R3 in Lung Cancer: From Metabolism to Therapeutics

Lung cancer is the top cause of cancer-related deaths globally, with most patients having a form known as non-small cell lung cancer (NSCLC - a type of lung cancer that grows and spreads more slowly than other types). Despite progress in understanding the genetic shifts in lung cancer, diagnosing and treating this disease still pose challenges. This study focused on a protein named Cytochrome b5 reductase 3 (CYB5R3 - a protein involved in multiple biological functions, including energy production and fat metabolism). The research discovered that CYB5R3 presence is reduced in lung cancer and that an increase in CYB5R3 can cause cancer cells to die. This implies that CYB5R3 might act as a tumor suppressor (a gene that regulates cell division and prevents tumor formation) and could be a potential treatment target for lung cancer. "This summary was initially drafted using artificial intelligence, then revised and fact-checked by the author."

## Introduction

Lung cancer is the leading cause of cancer-related mortality worldwide and has a 5-year survival rate of less than 20% [1, 2]. Lung cancer is histologically classified into two major types: small cell lung cancer (SCLC) and non-small cell lung cancer (NSCLC). NSCLC is the most prevalent subtype, accounting for 80–85% of lung cancer cases [3]. Genomic studies have revealed multiple genetic alterations in oncogenes and tumor suppressor genes (TSGs) in lung cancer [4, 5]. Although genetic alterations in TP53, EGFR, EML4-ALK, PIK3CA, and KRAS have been identified as biomarkers of lung cancer, difficulties remain in diagnosing, predicting the prognosis of, and treating lung cancer patients. Therefore, novel therapeutic targets must be developed to improve lung cancer diagnosis and treatment.

Cytochrome b5 reductase 3 (CYB5R3) catalyzes one-electron transfer from NADH to electron acceptors such as cytochrome b5 or coenzyme Q, resulting in the production of NAD^+^ [6]. CYB5R3 has two isoforms: soluble and membrane-bound. The soluble isoform expressed in erythrocytes has a truncated N-terminal binding domain and reduces methemoglobin [7], and the membrane-bound isoform expressed in most cells is anchored to the mitochondrial outer membrane, endoplasmic reticulum (ER), and plasma membrane. Membrane-bound CYB5R3 participates in coenzyme Q (CoQ) reduction [8], heme iron reduction [9, 10], fatty acid elongation and desaturation [11, 12], cholesterol biosynthesis [13], drug metabolism [14, 15], and aging [6, 12]. CYB5R3 is a reductase of soluble guanylate cyclase (sGC) in vascular smooth muscle cells that regulates cGMP production, protein kinase G signaling, and hypertension [10, 16]. CYB5R3 is transcriptionally regulated by FOXO3a and Nrf2 and controls both nutrient and oxidative stress responses [17]. CYB5R3 is a target of FoxO1 in β-cells and links mitochondrial dysfunction to β-cell failure [18], and it also promotes cell colonization and metastasis in estrogen receptor-negative breast cancer [19]. However, the functions and mechanisms of CYB5R3 in cancer biology have not yet been explored.

The ER plays key roles in protein folding, protein transport, calcium homeostasis, and lipid synthesis. Alterations in cellular processes, such as protein folding and calcium regulation, lead to the activation of the unfolded protein response (UPR), a prosurvival response to restore normal ER function; however, prolonged ER stress ultimately triggers cell death [20–22]. UPR activation is mediated by three stress sensors: the transmembrane receptors protein kinase RNA-like ER kinase (PERK), inositol-requiring enzyme 1 alpha (IRE1α) and activating transcription factor 6 (ATF6). These stress sensors bind to the ER chaperone GRP78 (also known as BiP) under nonstress conditions. ER stress, such as that induced by the accumulation of unfolded proteins, activates the UPR by dissociating stress sensors from GRP78. Activated PERK phosphorylates eukaryotic translation initiation factor-2 (eIF2), thereby inhibiting protein translation and increasing the expression of activating transcription factor-4 (ATF4) and its downstream target C/EBP-homologous protein (CHOP; encoded by *DDIT3*), which are involved in ER stress-induced apoptosis [23]. Similar to PERK, IRE1α splices the transcription factor X box-binding protein 1 (XBP1), and the spliced form of XBP1 induces the expression of genes involved in the UPR. When the recovery of ER function fails, active IRE1α recruits TNF receptor-associated factor 2 (TRAF2) and apoptosis signal-regulating kinase-1 (ASK1), which activates Jun-N-terminal kinase (JNK) and induces apoptosis [24, 25]. The transcription factor ATF6 is cleaved by the endopeptidases S1P and S2P in the Golgi apparatus, and cleaved ATF6 induces UPR targets, including GRP78 and XBP1 [23].

In this study, we provide evidence that CYB5R3 overexpression induces ER stress by promoting ADP-ribosylation of PERK and IRE1α, resulting in apoptosis in lung cancer cells. Our findings suggest that CYB5R3 functions as a tumor suppressor and can be utilized in the development of anti-lung cancer drugs.

## Material and methods

### Reagents and antibodies

Sulforhodamine B, tunicamycin, and GSH-EE were purchased from Sigma‒Aldrich (St. Louis, MO, USA). A genomic DNA kit was obtained from Promega (Madison, WI, USA). The anti-β-Tubulin (#2128), anti-ATF3 (#18665), anti-GADD45A (#4632), anti-ATF6 (#65880), anti-CHOP (#2895), anti-GRP78 (#3177), anti-XBP1 (#40435), anti-IRE1α (#3294), anti-DR5 (#8074), anti-Bax (#2772), anti-ATF4 (#11815), anti-Puma (#4976), anti-PERK (#3192), anti-p-eIF2α (#9721), anti-eIF2α (#9722), anti-p-JNK (#9251), anti-JNK (#9252), anti-cytochrome c (#4272), anti-COXIV (#4850), anti-Calnexin (#2679), anti-PARP1 (#9542), anti-Poly/Mono-ADP Ribose (#83732), anti-Caspase 3 (#9662), anti-Caspase 8 (#9746), and anti-Caspase 9 (#9502) antibodies were obtained from Cell Signaling Technology (Beverly, MA, USA). The anti-PARP16 antibody (ab154510) was obtained from Abcam (Cambridge, UK), the anti-Sestrin2 antibody (10795-1-AP) was obtained from Proteintech (Rosemont, IL, USA), the anti-CYB5R3 antibody (BS-12162R) was obtained from Bioss Antibodies Inc. (Woburn, MA, USA), the anti-GAPDH antibody (LF-P-A0212) was obtained from AbFrontier (Seoul, Korea), the anti-ARTC1 (SAB1300652) and anti-Flag (F1804) antibodies were obtained from Sigma‒Aldrich, and the mouse anti-FITC (sc-2010) and rabbit anti-rhodamine (sc-2492) antibodies were obtained from Santa Cruz Biotechnology (Dallas, TX, USA).

### Cell culture and transfections

Human lung fibroblasts (IMR-90 and WI-38) and NSCLC cells (NCI-H1299 (H1299), NCI-H1703 (H1703), NCI-H226 (H226), NCI-H23 (H23), NCI-H460 (H460), NCI-H2009 (H2009), HCC827, and A549) were purchased from the Korean Cell Line Bank (Seoul, Korea) and the KRIBB Cell Line Bank (Daejeon, Korea). IMR-90, WI-38, and A549 cells were cultured in Dulbecco’s modified Eagle’s medium (DMEM), while H226, H460, H2009, HCC827, H23, H1703, and H1299 cells were cultured in RPMI-1640 medium supplemented with 10% fetal bovine serum (FBS) and penicillin/streptomycin (Invitrogen, Carlsbad, CA, USA). All cell lines were tested for *Mycoplasma* contamination using an e-Myco^TM^ VALiD *Mycoplasma* PCR Detection Kit (iNtRON Biotechnology, Gyeonggi-Do, Korea).

siRNA pools (ON-TARGETplus SMARTpool, containing four different siRNAs that target a single gene) against ATF3 (L-008663), DDIT3/CHOP (L-004819), SESN2 (L-019134), ERN1/IRE1 (L-004951), EIF2AK3/PERK (L-004883), ATF6 (L-009917), ATF4 (L-005125), JNK1 (L-003514), JNK2 (L-003505), JNK3 (L-004324), ARTC1 (L-010387), and PARP16 (L-020837), and a nontargeting control (D-001810-10-05) were obtained from Dharmacon (Lafayette, CO, USA). The CYB5R3 siRNA was purchased from Bioneer Corporation (Daejeon, Korea). The target sequences were as follows: siCYB5R3 #1, 5′-GUUUACUUCAAGGACACCCAU-3′; siCYB5R3 #2, 5′-AGAACCUCAGCAUUUCCUU-3′; siScrambled: 5′-CCUACGCCACCAAUUUCGU-3′. Cells were transfected with siRNAs (40 nM) using an electroporation instrument (Neon, Invitrogen) according to the manufacturer’s instructions.

### Adenoviruses

The adenoviral CYB5R3-Flag (Ad-CYB5R3) vector and empty vector control (EV) were obtained from Vigene Biosciences (Rockville, MD, USA). AD293 cells were reinfected with viral stocks to replicate the virus, and viral particles were then purified by double cesium chloride gradient ultracentrifugation. The infectious viral particles in the cesium chloride gradient were collected, dialyzed against 10 mM Tris (pH 8.0), 2 mM MgCl_2_, and 5% sucrose solution, and stored in a deep freezer. Viral titers were determined using the Adeno-X^TM^ Rapid Titer Kit (Takara Bio USA, Inc., Mountain View, CA, USA) according to the manufacturer’s protocol. Cells were infected with adenoviruses at a multiplicity of infection (MOI) of 100.

### Animal experiments

All mouse experiments were conducted in accordance with a protocol approved by the Institutional Animal Care and Use Committee. Tumor formation was induced in C57BL/6 (Orient Bio, Gyeonggi-Do, Korea) mice by 10 weekly intraperitoneal (IP) injections of 0.5 mg/g urethane (ethyl carbamate, Sigma‒Aldrich) [26]. The mice were sacrificed eight months after urethane injection. Lungs were fixed, embedded, stained with hematoxylin and eosin (H&E), and analyzed under an Olympus microscope (BX51, Tokyo, Japan).

For the mouse xenograft assay, tumors were established by subcutaneously injecting NCI-H1299 cells (5 ×10^6^ cells/mouse) into six-week-old BALB/c female nude mice (Orient Bio). Tumor volumes were estimated using the following formula: (length (mm) × width (mm) × height (mm))/2. When the average tumor volume reached 100 cm^3^, the mice were randomized into two groups (*n* = 8 mice per group), and adenoviral EV or CYB5R3 vector (1 ×10^9^ pfu per mouse) was administered intratumorally every three days for a total of three treatments. The mice were euthanized on Day 18, and the tumors were weighed.

### Generation of CYB5R3 knockout mice

CYB5R3 knockout mice were generated by GH Bio (Daejeon, Korea). A single-guide RNA (sgRNA) targeting the N-terminal region of CYB5R3 was designed using the ZiFiT (http://zifit.partners.org/ZiFiT/) program. The spacer sequences of the sgRNAs were as follows: sgRNA1, 5′-CTTGATGTCGGGGTTCTCGA-3′; sgRNA2, 5′-AGACTCCGAGTAGCTGTTCC-3′; and sgRNA3, 5′-TCTGAGGCTCATCGACAAGG-3′. The two complementary oligonucleotides of each sgRNA were annealed, and the sgRNAs were cloned and inserted into the pT7-gRNA vector, a vector designed for synthesizing sgRNAs [27]. In vitro transcription of the sgRNA for CYB5R3 and purification of short RNAs was performed using a MEGAshortscript T7 kit (ThermoScientific, Waltham, MA, USA) according to the manufacturer’s instructions. Microinjection was performed in fertilized eggs from C57BL/6N (Orient Bio) mice. A mixture of the sgRNA (100 ng/µl) and the Cas9 protein (80 ng/µl) (ToolGen Inc., Seoul, Korea) was injected into the cytoplasm of pronuclear-stage embryos. Injected embryos were cultured in medium overnight prior to transfer into pseudopregnant mice (ICR strain). Genomic DNA was extracted from the tails of the progeny mice and subjected to PCR using the following primer pair: forward primer, 5′-TGGAGTTCTCTGGTCAAGGC-3′; reverse primer, 5′-TTGGCTGTCATTGTGCCTGA-3′. PCR products were analyzed by agarose gel electrophoresis and sequencing to confirm the identity of every allele.

### Immunohistochemistry

Human tissue arrays were obtained from US Biomax (Rockville, MD, USA). Immunohistochemical (IHC) analysis was performed as previously described [28]. Briefly, slides were incubated overnight with an anti-CYB5R3 antibody. After washing, the slides were incubated with a biotinylated HRP complex (Vector Laboratories, Burlingame, CA, USA), and 3,3’-diaminobenzidine (DAB substrate kit; Vector Laboratories) was used for color development. Then, the slides were stained with hematoxylin and eosin (H&E).

### Cell viability and IncuCyte system

Cell viability was determined using a sulforhodamine B assay, as previously described [29]. A cell growth inhibition assay (3000 cells/well in a 96-well plate) was performed in cells transduced with the adenoviral CYB5R3 vector and EV for 72 h. Cell death was analyzed with the CellPlayer reagent-based annexin V (red) or caspase-3/7 (green) reporter according to the manufacturer’s protocols (IncuCyte ZOOM System, Essen Bioscience, Ann Arbor, MI, USA). Green and red fluorescence images and phase contrast images were acquired at 2 h intervals using a 10× objective lens.

### RNA sequencing (RNA-seq) analysis

Total RNA was isolated using an mRNA isolation kit (QIAGEN, Valencia, CA, USA) according to the manufacturer’s instructions. Four micrograms of RNA was prepared using the TruSeq Stranded mRNA LT Sample Prep Kit. The library was sequenced using the Illumina NovaSeq 6000 system (Illumina, San Diego, CA, USA) to generate 100 bp paired-end reads. The sequencing reads were mapped to the human genome (GRCh38/hg38) using STAR (v.2.7.3a), and gene expression was quantified using the count module in STAR. Differentially expressed genes (DEGs) were identified from the RNA-seq count data using the edgeR package (v.3.32.0).

### Quantitative reverse transcription–polymerase chain reaction (QRT-PCR)

Two micrograms of total isolated RNA was reverse transcribed into cDNA using a TOPscript RT Kit (Enzynomics, Daejeon, Korea) according to the manufacturer’s protocol. QPCR was performed using the SYBR Green Master Mix Kit (QIAGEN) on a Rotor-Gene Q system (QIAGEN). The following primers were obtained from Bioneer Corporation: DUSP1 (P199349), DUSP2 (P259112), DUSP5 (P197350), DUSP10 (P210086), PPP1R15A (P192005), DDIT3 (P225750), ERN1 (P298108), XBP1 (190450), GADD45A (P230161), BBC3 (P150935), TNFRSF10B (P195586), TRAF1 (P141876), ATF3 (P292769), KLF4 (P154272), and SESN2 (P153243). All reactions were performed in triplicate, and mRNA expression was normalized to β-actin (QIAGEN) expression as an internal control.

### Metabolomic analysis

Metabolomic analysis was performed by Human Metabolome Technologies, Inc. (Tsuruoka, Japan) using the CARCINOSCOPE (C-SCOPE) platform. H1299 cells (2 ×10^6^) were seeded into a 100 mm dish the day before the assay and incubated with the adenoviral empty vector control (EV) or CYB5R3-expressing vector for 24 or 36 h (*n* = 3 samples/group). Metabolite extraction was performed according to the manufacturer’s protocol (HMT), as described previously [30]. Absolute quantitative analysis of 116 metabolites (54 cations and 62 anions) was performed on 12 samples of harvested cells using capillary electrophoresis–mass spectrometry (CE–MS).

### Immunoprecipitation and immunoblot analysis

Cells were lysed with 1× RIPA buffer (Millipore, Temecula, CA, USA) containing 1 mM Na_3_VO_4_, 1 mM sodium fluoride, 1 mM PMSF, and a protease inhibitor cocktail (Roche, Basel, Switzerland), and the protein concentrations in the lysates were quantified using a BCA protein assay kit (Thermo Scientific, 23227). For immunoprecipitation, 1 mg of each lysate was incubated with 2 μg of the indicated antibody or normal rabbit IgG at 4 °C overnight and then with 20 μl of Protein A/G PLUS Agarose (sc-2003, Santa Cruz Biotechnology) at 4 °C for 1 h. The agarose was washed three times with wash buffer (0.1% NP40 in phosphate-buffered saline (PBS)).

Mitochondrial isolation was performed using H1299 cells and a Mitochondrial Isolation Kit according to the manufacturer’s protocol (Thermo Scientific, 89874). The lysates were then subjected to immunoblotting using specific antibodies. Immunoblot signals were detected using an Enhanced Chemiluminescence (ECL) Kit (Millipore).

### Immunofluorescence and DCF-DA staining

Cells were plated onto a 8-well microslide plate (ibidi Inc., Fitchburg, WI, USA), fixed in 4% paraformaldehyde for 30 min, permeabilized in PBS with 0.3% Triton X-100 for 10 min, and blocked with 3% bovine serum albumin for 1 h at 25 °C. The cells were incubated overnight with appropriate antibodies. For mitochondrial tracker staining, cells were incubated with the tracker dye for 30 min and washed with PBS. The cells were incubated with 10 μM H2DCFDA (Invitrogen) for 30 min and washed with PBS to measure ROS generation. Finally, the cells were counterstained with DAPI for 10 min to label nuclei and were then analyzed using a confocal microscope (LSM5 Live DuoScan, Carl Zeiss, Stuttgart, Germany).

### H_2_O_2_ measurement

H_2_O_2_ was measured using the ROS-Glo H_2_O_2_ assay according to the manufacturer’s protocol (Promega, G8820). Cells were plated into a 96-well plate (Thermo Scientific, 136101) at a density of 10,000 cells/well and transduced with the adenoviral CYB5R3 vector and EV for 24 h. Cells were incubated with H_2_O_2_ substrate solution for 6 h and with ROS-Glo detection solution for 20 min. Luminescence was measured with a multimode microplate reader (BioTek Synergy HTX, Agilent, Santa Clara, CA, USA).

### Generation of knockout cells with the CRISPR‒Cas9 strategy

sgRNAs were cloned and inserted into the lentiCRIPR V2 plasmid as previously reported [31]. Briefly, each sgRNA oligonucleotide annealed with the T4 PNK enzyme (#M0201, NEB, Ipswich, MA, USA) was cloned and inserted into the lentiCrispr V2 vector digested with BsmBI (#R0739, NEB). To generate lentiviruses, 293T cells in 100-mm tissue culture dishes were transfected with 6 μg of each sgRNA along with 3.34 μg of the pLP1 and 2.2 μg of the pLP2 packaging plasmids and 3.34 μg of the VSVg envelope plasmid using polyethyleneimine. Viral particles were produced and used to transduce cells, and puromycin (3–10 μg/mL) selection was then performed. Gene targeting was confirmed using immunoblot analysis. The target sequences of the sgRNAs are listed below. Negative control (sgNeg): 5′-GAAGATGGGCGGGAGTCTTC-3′, CYB5R3 #1: 5′-AGGCATCACCCCGATGCTGC-3′, CYB5R3 #2: 5′-GTGTATAGGGCCGGACGACC-3′, CYB5R3 #3: 5′-TCCCGGTCGATGAGCCGCAG-3′, and CYB5R3 #4: 5′-GAAGACGAAGCAGCGCTCCG-3′.

### Statistical analysis

All data represent the results of at least three independent experiments. The results are presented as the means ± SDs. Statistical analyses were performed by unpaired, two-tailed Student′s *t* test or two-way ANOVA with correction for multiple comparisons using GraphPad Prism ver 9.0 software (GraphPad Software, Boston, MA, USA). The statistical significance threshold was set at *p* < 0.05.

## Results

### CYB5R3 expression is downregulated in lung cancer

**Fig. 1:**
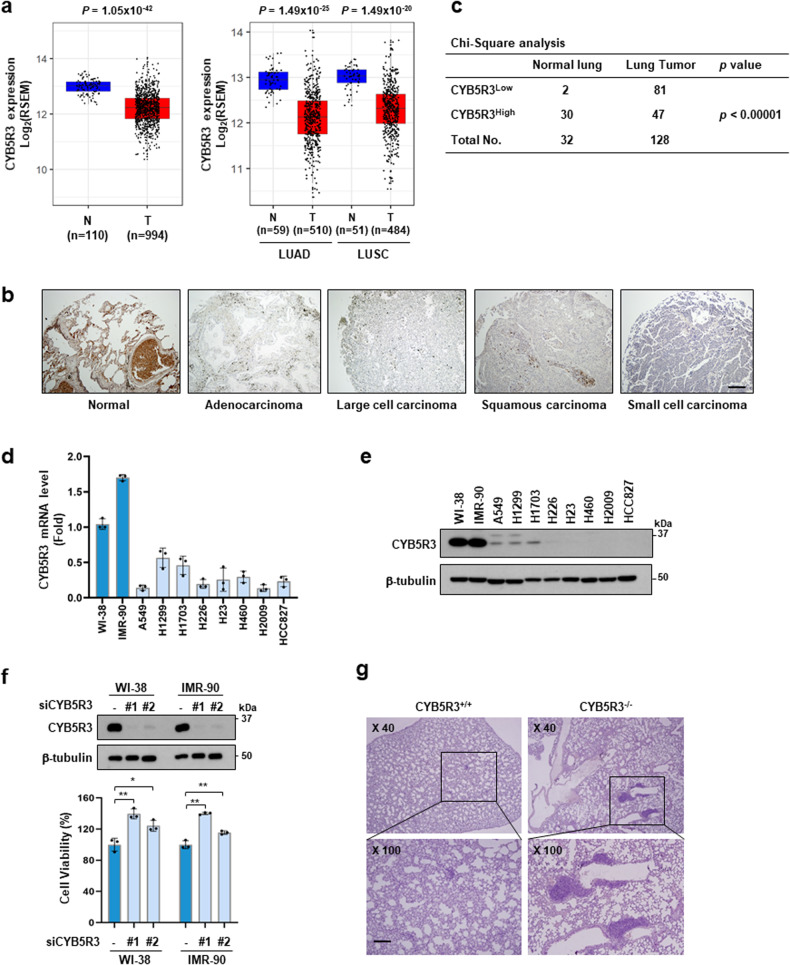
CYB5R3 is downregulated in lung cancer. **a** CYB5R3 expression profiles across lung tumor samples (T) and corresponding normal tissues (N) in the TCGA database. The expression data were collected from the TCGA cohort via cBioPortal (http://cbioportal.org) and were then visualized by ggplot2 (version 3.3.6). LUAD; lung adenocarcinoma, LUSC; lung squamous carcinoma. All statistical analyses were performed using R software. **b** IHC analysis of CYB5R3 in normal lung tissue and lung cancer patient tissue. Representative images of CYB5R3 staining in the tissue array are shown. Scale bar, 100 μm. **c** CYB5R3 expression levels in normal lung tissues and lung cancer tissues. CYB5R3^Low^ indicates negative (0) or weak (1) IHC staining, and CYB5R3^High^ indicates moderate (2) or strong (3) IHC staining. CYB5R3 expression in two human lung fibroblast and eight lung cancer cell lines. mRNA (**d**) and protein levels (**e**) were measured by quantitative RT‒PCR and immunoblot analysis, respectively. **f** Effects of CYB5R3 siRNA on the protein level of CYB5R3 (upper panel) and cell viability (lower panel) in WI-38 and IMR-90 cells. The values are presented as the mean ± SD of three independent experiments. *P < 0.05, **P < 0.01. **g** H&E staining of lung tissues in CYB5R3^+/+^ and CYB5R3^−/−^ mice 8 months after IP injections of 0.5 mg/g urethane (*n* = 3 mice per group). Scale bar, 100 μm.

To investigate the relevance of CYB5R3 to tumorigenesis, we analyzed the CYB5R3 expression profile in cohorts in The Cancer Genome Atlas (TCGA) obtained via cBioPortal (http://cbioportal.org) using R software. TCGA data analysis revealed that CYB5R3 mRNA expression was significantly decreased in 13 of 24 subtypes of cancer tissues compared to the corresponding normal tissues in patients represented in TCGA (Supplementary Fig. 1a). CYB5R3 expression was downregulated in both lung adenocarcinoma (LUAD) and lung squamous cell carcinoma (LUSC) (Fig. 1a). Immunohistochemical (IHC) analysis was performed using a human tissue array containing 32 normal lung tissues or adjacent normal lung tissues and 128 lung cancer samples. Consistent with the analysis of public data, IHC analysis revealed that CYB5R3 expression in lung cancer tissues was lower than that in normal tissues (Fig. 1b and Supplementary Fig. 1b). We also found that 91% (29 of 32) of the normal lung tissues exhibited high CYB5R3 expression, while only 19% (24 of 128) of the lung cancer tissues showed high CYB5R3 expression (Fig. 1c), indicating that CYB5R3 expression is associated with lung carcinogenesis. However, the expression levels of CYB5R1 and CYB5R2 were not downregulated in LUAD tissue (Supplementary Fig. 2a, b). Neither CYB5R1 nor CYB5R2 was detected in human lung cancer cells (Supplementary Fig. 2c).

To examine the expression pattern of CYB5R3 in human lung cancer cells, we measured CYB5R3 mRNA levels using quantitative RT‒PCR in two human lung fibroblast lines and eight NSCLC cell lines. The mRNA levels of CYB5R3 in the NSCLC cell lines A549, H1299, H1703, H226, H23, H460, H2009, and HCC827 were lower than those in the normal lung fibroblast lines WI-38 and IMR-90 (Fig. 1d). Similarly, the CYB5R3 protein was relatively abundant in WI-38 and IMR-90 cells compared to the NSCLC cell lines (Fig. 1e). To examine the effect of CYB5R3 depletion on cell growth, we performed CYB5R3 knockdown using two different siRNAs targeting CYB5R3 in WI-38 and IMR-90 cells. CYB5R3 expression was significantly decreased in CYB5R3-knockdown cells (Fig. 1f, upper panel). Compared with siScrambled (siScr) transfection, CYB5R3 knockdown promoted the growth of WI-38 and IMR-90 cells (Fig. 1f, lower panel).

### CYB5R3 is functionally associated with tumor suppression in a mouse model

We evaluated the role of CYB5R3 in tumorigenesis using an in vivo mouse model (Supplementary Fig. 3). First, we examined the expression profile of CYB5R3 in mouse tissues and found that it was abundantly expressed in the lungs, liver, testes, and ovaries in mice (Supplementary Fig. 3a). However, unlike in mouse primary lung fibroblasts, the CYB5R3 protein was not detected in Lewis lung carcinoma (LLC) cells, a murine lung cancer cell line (Supplementary Fig. 3b). CYB5R3 knockout (KO) mice were generated using the CRISPR‒Cas9 system (Supplementary Fig. 3c). We observed that CYB5R3 KO mice were healthy and exhibited normal fertility. No significant difference was observed in body weight between the CYB5R3 KO and WT groups (Supplementary Fig. 3d). Hematological analysis revealed that CYB5R3 KO mice had fewer white blood cells than WT mice (Supplementary Table 1). As expected, CYB5R3 protein expression was not detected in the lungs, liver, spleen, or kidneys in CYB5R3^−/−^ mice (Supplementary Fig. 3e). We then evaluated the incidence of lung cancer in CYB5R3^−/−^ mice eight months after treatment with urethane. H&E staining showed a higher incidence of tumors in the lung tissue in CYB5R3^−/−^ mice than in CYB5R3^+/+^ mice (Fig. 1g). In the lung metastasis assay using LLC cells, the incidence and size of tumors in the lung tissues were considerably increased in CYB5R3^−/−^ mice compared with CYB5R3^+/+^ mice (Supplementary Fig. 3f, g). These results suggest that CYB5R3 acts as a tumor suppressor in lung cancer.

### CYB5R3 overexpression leads to apoptosis in lung cancer cells

**Fig. 2:**
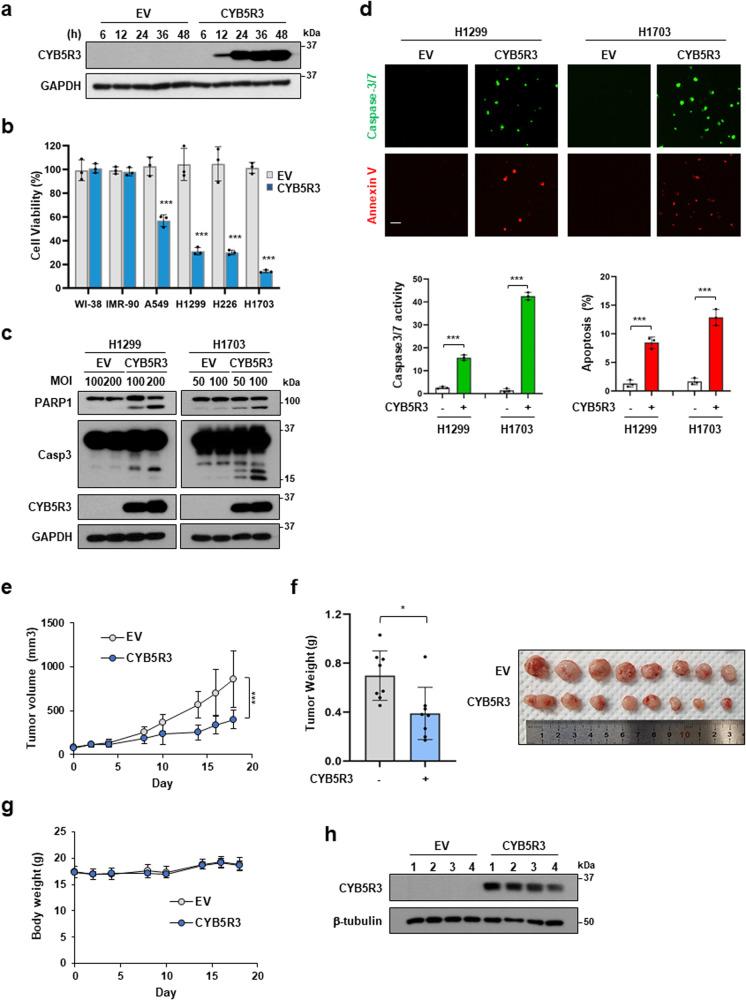
CYB5R3 overexpression induces cell death in lung cancer cells in vitro and in vivo. **a** Cells were infected with EV or CYB5R3 adenovirus at an MOI of 100 for the indicated times, and cell lysates were analyzed by immunoblotting. **b** Cells were infected with EV or CYB5R3 adenovirus at an MOI (multiplicity of infection) of 100 for 72 h. Cell viability was measured by an SRB assay. The data are presented as the mean ± SD of three independent experiments. **c** Cleavage of PARP1 and caspase-3 in CYB5R3-infected H1299 and H1703 cells. **d** Caspase 3/7 activation and annexin staining in CYB5R3-infected H1299 cells. Representative images are shown. Scale bar, 100 μm. **e** Tumor volume was measured at 2-3-day intervals in xenografted H1299 mice (*n* = 8 mice/group). **f** Xenograft tumor weights (*n* = 8 mice/group). **g** Body weights (*n* = 8 mice/group). **h** CYB5R3 protein level in H1299 xenograft tissues. The data are presented as the means ± SDs. Statistical analysis was performed by two-way ANOVA with post hoc Tukey’s test (**e**). *P < 0.05, **P < 0.01, ***P < 0.001.

To confirm the tumor-suppressive role of CYB5R3 in human lung cancer cells, we investigated the effects of CYB5R3 overexpression using Ad-CYB5R3, an adenovirus that expresses CYB5R3. CYB5R3 protein expression was detectable within 12 h and showed the highest values 24 h after infection with CYB5R3 (Fig. 2a). CYB5R3 overexpression dramatically inhibited the growth of A549, H1299, H226, and H1703 cells compared to EV transduction but did not inhibit the growth of WI-38 and IMR-90 cells (Fig. 2b). Cleavage of PARP1 and capase-3 was detected in H1299 and H1703 cells infected with Ad-CYB5R3 (Fig. 2c). A caspase 3/7 activity assay and annexin V staining showed that CYB5R3 significantly induced apoptosis in lung cancer cells (Fig. 2d). To investigate the effect of CYB5R3 on tumor growth in vivo, we performed a xenograft assay using H1299 cells. Compared with EV-treated mice, CYB5R3-treated mice exhibited reductions of 53.3% and 44.2% in tumor volume and weight, respectively, without significant changes in body weight (Fig. 2e–g). Immunoblot analysis of the resected tumors revealed that the protein level of CYB5R3 was dramatically increased in CYB5R3-treated tumors compared to EV-treated tumors (Fig. 2h). These data imply that CYB5R3 functions as a tumor suppressor in lung cancer.

### CYB5R3 overexpression affects the transcriptional landscape of lung cancer cells

**Fig. 3:**
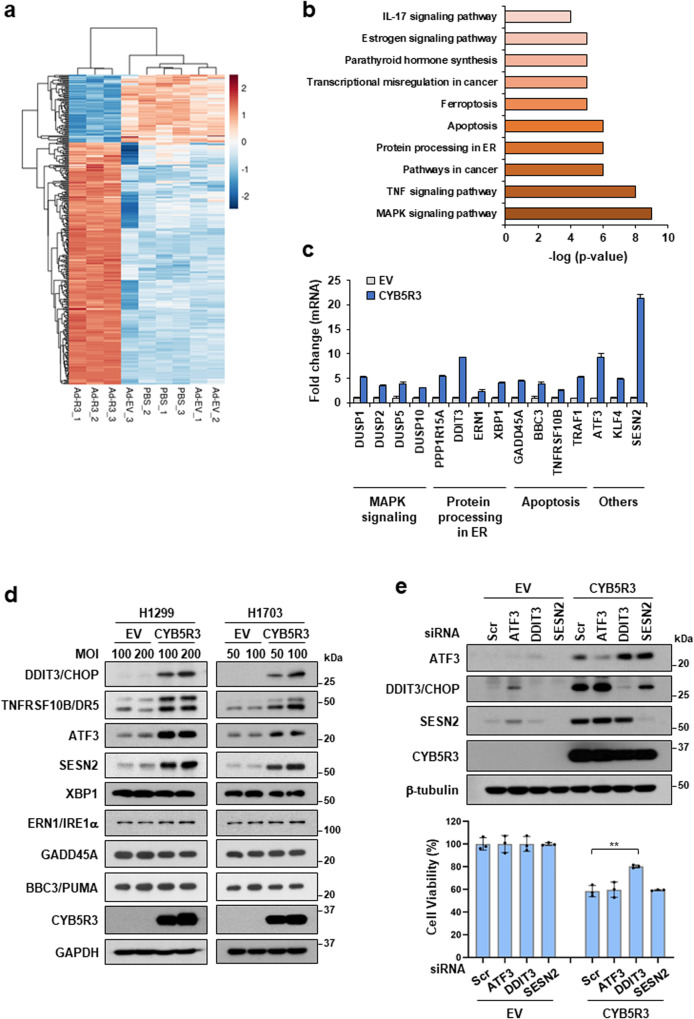
CYB5R3 drives changes in gene expression profiles. **a**–**c** RNA-seq analysis in H1299 cells infected with EV (Ad-EV) and CYB5R3 (Ad-CYB5R3). **a** Heatmap showing the differentially expressed genes (DEGs) from three independent samples treated with CYB5R3 compared with EV or PBS. **b** KEGG pathway enrichment analysis of the DEGs. The top twenty pathways are shown. **c** QPCR analysis of the indicated genes in H1299 cells infected with CYB5R3 for 24 h. **d** Immunoblot analysis of proteins with altered expression in H1299 or H1703 cells infected with CYB5R3 for 24 h. **e** H1299 cells were transfected with the indicated siRNA and infected with CYB5R3 for 48 h. Cell viability was measured by an SRB assay (lower panel). Immunoblot analysis was performed to measure the indicated protein levels (upper panel). The experiments were repeated three times. The data are shown as the means ± SDs. Student′s *t* test. **P < 0.01.

To investigate the molecular mechanism by which CYB5R3 induces cancer cell death, we analyzed the gene expression pattern induced by CYB5R3 overexpression in H1299 cells. RNA sequencing analysis revealed 248 upregulated and 69 downregulated genes in cells infected with Ad-CYB5R3 compared with those infected with EV (Fig. 3a). Genes involved in mitogen‑activated protein kinase (MAPK) signaling, the TNF signaling pathway, pathways in cancer, protein processing in ER, and the apoptosis pathway were upregulated in CYB5R3-overexpressing cells (Fig. 3b). The RNA sequencing data were verified using real-time PCR. The upregulated genes included DUSP1, DUSP2, DUSP5, and DUSP10 in MAPK signaling; DDIT3, ERN1, PPP1R15A, and XBP1 in protein processing in the ER; BBC3, GADD45A, TNFRSF10B, and TRAF1 in apoptosis; and ATF3, KLF4, and SESN2 in other pathways (Fig. 3c). Interestingly, the levels of the CHOP (DDIT3), DR5 (TNFRSF10B), ATF3, and SESN2 proteins were dramatically increased in CYB5R3-overexpressing H1299 and H1703 cells following Ad-CYB5R3 infection (Fig. 3d). In contrast, the expression of the XBP1, IRE1α (ERN1), PUMA (BBC3), and GADD45A proteins remained unchanged. To evaluate how genes upregulated by CYB5R3 overexpression are involved in cell death, we transfected siRNAs against ATF3, DDIT3, or SESN2 into H1299 cells. DDIT3 silencing overcame CYB5R3-induced cell death (Fig. 3e, lower panel). The knockdown efficiency of each siRNA is shown in Fig. 3e (upper panel). These data suggest that CHOP (DDIT3) induction is critical for CYB5R3-induced cell death in lung cancer cells.

### CYB5R3 triggers ER stress via the PERK-ATF4 and IRE1α-JNK pathways

**Fig. 4:**
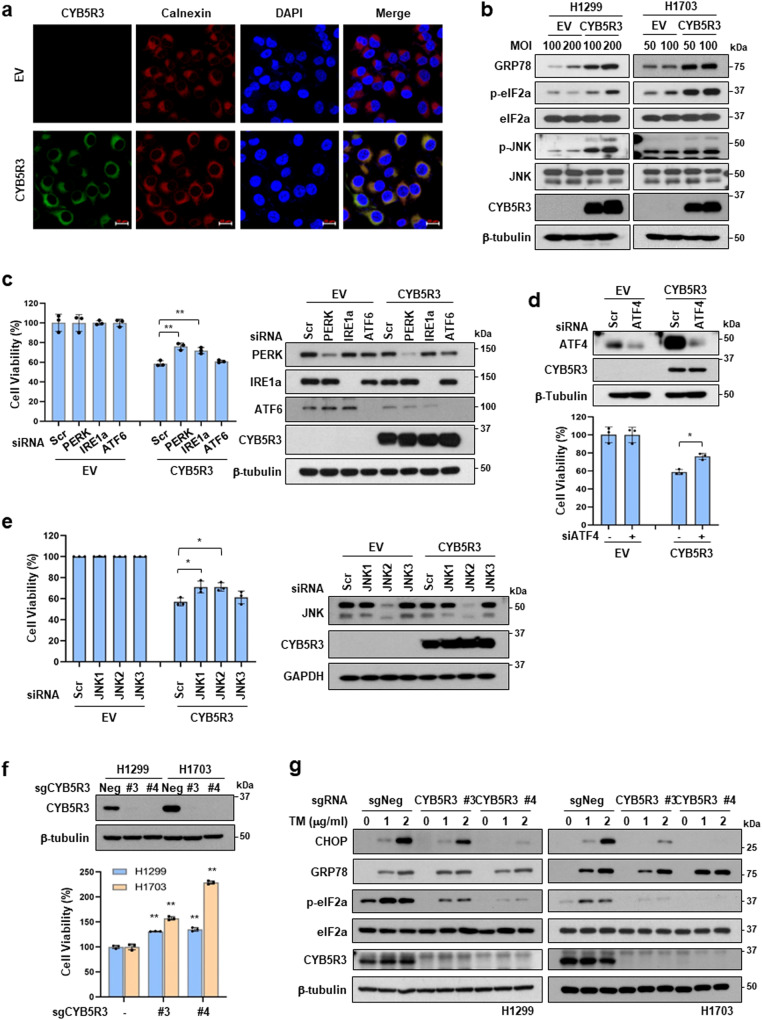
CYB5R3 induces ER stress. **a** Immunofluorescence staining of CYB5R3 (green) and calnexin (red) was carried out in H1299 cells infected with EV or CYB5R3. DAPI (blue) was used for nuclear staining. Scale bar, 20 μm. **b** Protein levels of ER markers in H1299 cells infected with EV or CYB5R3 for 24 h. **c** H1299 cells were transfected with the indicated siRNA and infected with CYB5R3 for 48 h. A cell viability assay (left panel) and immunoblot analysis (right panel) were performed. **d** H1299 cells were transfected with siRNA against ATF4 and infected with CYB5R3 for 48 h. Cell viability (lower panel) or immunoblot analysis (upper panel) was performed. **e** H1299 cells were transfected with the indicated siRNAs and infected with CYB5R3 for 48 h. A cell viability assay (left panel) and immunoblot analysis (right panel) were performed. **f** Protein level of CYB5R3 (upper panel) and cell viability (lower panel) in H1299 and H1703 cells with CRISPR/Cas9-mediated CYB5R3 knockout. **g** Protein levels of ER markers in tunicamycin-treated CYB5R3-knockout H1299 and H1703 cells. The values are presented as the mean ± SD of three independent experiments. *P < 0.05, **P < 0.01.

Since CHOP (DDIT3) expression is a major hallmark of ER stress and induces ER stress-mediated apoptosis [32], we examined the relationship between CYB5R3 overexpression and ER stress. Immunofluorescence staining showed that CYB5R3 colocalized with calnexin, an ER marker, in CYB5R3-overexpressing H1299 cells (Fig. 4a). CYB5R3 also localized in mitochondria, consistent with the results of previous studies [19, 33] (Supplementary Fig. 4).

To investigate whether CYB5R3 regulates ER function, we analyzed the expression levels of the ER stress sensors PERK, IRE1α, and ATF6 and their downstream targets. Similar to CHOP induction (Fig. 3d), CYB5R3 overexpression increased the expression of ER stress signaling proteins, such as GRP78, p-eIF2α, and p-JNK, which triggered apoptosis in both H1299 and H1703 cells (Fig. 4b). To assess the pathways involved in CYB5R3-induced cell death, we performed gene knockdown assays using siRNAs against PERK, IRE1α, or ATF6 in H1299 cells. Importantly, silencing of PERK or IRE1α but not ATF6 overcame CYB5R3-induced cell death (Fig. 4c). Moreover, deletion of ATF4, which is a downstream target of PERK, or deletion of the JNK isoforms JNK1 and JNK2, which are downstream targets of IRE1α, restored CYB5R3-induced cell death (Fig. 4d, e).

To examine the interplay between CYB5R3 and ER stress, we generated CYB5R3 knockout H1299 and H1703 cells using the CRISPR‒Cas9 system. We used four different sgRNAs against CYB5R3 (sgCYB5R3) and found that sgCYB5R3 #3 and #4 completely suppressed CYB5R3 expression (Fig. 4f, upper panel). As expected, CYB5R3 knockout cells displayed a significant increase in growth compared to control (sgNeg) cells (Fig. 4f, lower panel), consistent with previous data from the normal lung fibroblasts WI38 and IMR-90 (Fig. 1f). Following treatment with tunicamycin, an ER stress-inducing antitumor agent, the levels of GRP78, CHOP, and p-eIF2α were increased in H1299-sgNeg and H1703-sgNeg cells, whereas the induction of CHOP and level of p-eIF2α were attenuated in H1299-sgCYB5R3 and H1703-sgCYB5R3 cells (Fig. 4g). These data suggest that CYB5R3-induced cell death is dependent on the PERK-ATF4 and IRE1α-JNK signaling pathways.

### CYB5R3 overexpression drives metabolic reprogramming

**Fig. 5:**
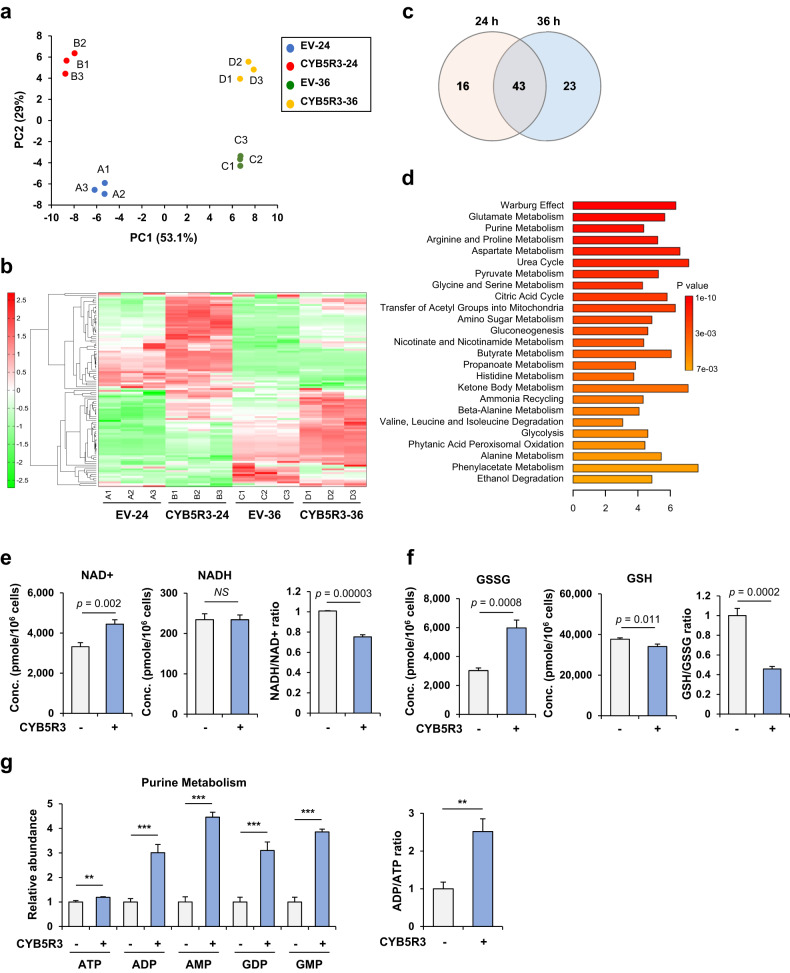
CYB5R3 leads to metabolic alterations. Metabolomic analysis in H1299 cells infected with EV or CYB5R3 at an MOI of 100 for 24 h or 36 h. **a** Principal component analysis (PCA) with three independent samples of the four groups infected with A. EV for 24 h, B. CYB5R3 for 24 h, C. EV for 36 h, or D. CYB5R3 for 36 h. **b** Heatmap showing the metabolites with statistically significant differences in abundance in three independent samples in CYB5R3-infected versus EV-infected cells. **c** Venn diagram showing the number of altered metabolites in cells infected with EV vs. CYB5R3 for 24 h or 36 h. **d** Enrichment analysis of the metabolites with statistically significant differences in abundance between cells infected with EV and CYB5R3 for 24 h. **e**–**g** Concentrations of altered metabolites in cells infected with CYB5R3 compared to EV for 24 h. *P < 0.05, **P < 0.01, ***P < 0.001.

Given that CYB5R3 functions in energy and lipid metabolism [12], we speculated that CYB5R3 leads to metabolic alterations. To investigate the metabolic changes in H1299 cells infected with Ad-CYB5R3, we performed metabolomic analysis using capillary electrophoresis and time-of-flight mass spectrometry (CE-TOFMS). Principal component analysis (PCA) revealed that CYB5R3 overexpression resulted in marked differences in metabolic signatures (Fig. 5a and Supplementary Table 2). Heatmap analysis revealed that the cells transduced with EV and the CYB5R3 vector were grouped into distinct metabolic clusters, and changes in 59 and 66 metabolites were observed in cells transduced with the CYB5R3 vector for 24 and 36 h, respectively, relative to those in cells transduced with EV (Fig. 5b). Subsequently, we conducted pathway enrichment analysis of CYB5R3-related metabolites using MetaboAnalyst 4.0 to comprehensively analyze the metabolic changes. We found that metabolites significantly increased by CYB5R3 expression were associated with the Warburg effect, glutamate, purine metabolism, arginine and proline metabolism, aspartate metabolism, urea cycle, glycine and serine metabolism, and the citric acid cycle (Fig. 5c, d). Redox homeostasis and purine metabolism were simultaneously altered in cells infected with CYB5R3 for 24 h and 36 h. Although the level of reduced glutathione (GSH) was decreased, the levels of oxidized GSH (GSSG), NAD^+^, AMP, and ADP were markedly increased 24 h after CYB5R3 transduction (Fig. 5e–g). These data suggested that CYB5R3 overexpression induces metabolic changes in lung cancer cells.

### CYB5R3 increases PARP16-mediated ADP-ribosylation of PERK and IRE1α

**Fig. 6:**
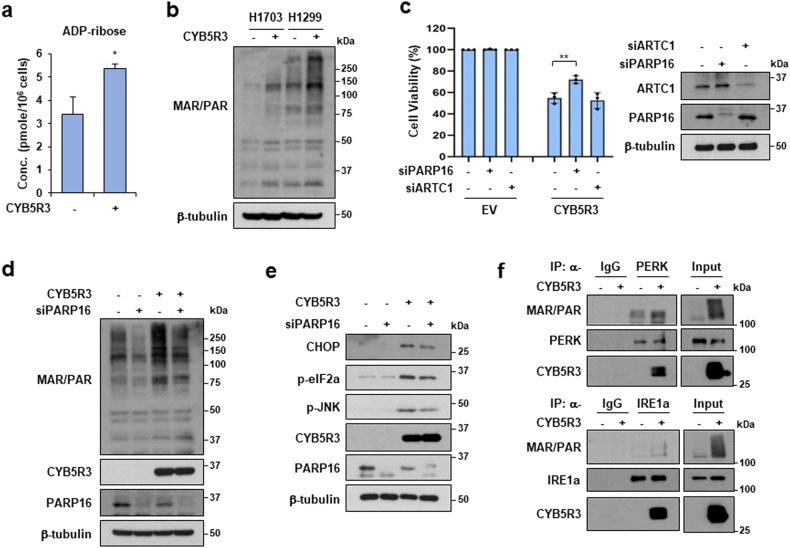
CYB5R3 promotes ADP-ribosylation by PARP16. **a** ADP-ribose level in H1299 cells infected with CYB5R3. **b** MAR/PAR levels in H1299 and H1703 cells infected with CYB5R3. **c** H1299 cells were transfected with the indicated siRNA and infected with CYB5R3 for 48 h. A cell viability assay (left panel) and immunoblot analysis (right panel) were performed. **d**, **e** H1299 cells were transfected with PARP16 siRNA and infected with CYB5R3 for 48 h. Immunoblot analysis was performed using the indicated antibodies. **f** H1299 cells were infected with EV or CYB5R3 for 24 h and immunoprecipitation was then performed with normal rabbit IgG, an anti-PERK antibody, or an anti-IRE1α antibody. The immunoprecipitates were analyzed by immunoblotting using the indicated antibodies.

Next, we explored how CYB5R3 activates PERK and IRE1α. NADH oxidation induced by CYB5R3 overexpression can affect NAD^+^-dependent signaling pathways and processes, such as ADP-ribosylation and protein deacetylation [34, 35]. Metabolomic analysis revealed that ADP-ribose levels were higher in CYB5R3-overexpressing cells than in control cells (Fig. 6a). A previous study demonstrated that ARTC1-mediated ADP-ribosylation of GRP78 is inactive, which activates the ER stress response [36]. Moreover, ER-resident PARP16 activates PERK and IRE1α via ADP-ribosylation in the ER [37]. To investigate whether ADP-ribosylation is involved in the activation of PERK and IRE1α induced by CYB5R3, we examined total ADP-ribosylation, including mono-ADP-ribosylation (MAR) and poly-ADP-ribosylation (PAR). Indeed, CYB5R3 overexpression increased overall ADP-ribosylation (Fig. 6b). We examined the effects of silencing ARTC1 and PARP16 on CYB5R3-induced cell death. Importantly, PARP16 depletion reduced CYB5R3-induced cell death (Fig. 6c). We found that the increase in ADP-ribosylation in CYB5R3-overexpressing cells was attenuated by PARP16 knockdown (Fig. 6d). Moreover, depletion of PARP16 decreased the protein levels of ER stress markers, such as CHOP, p-elF2a, and p-JNK, which were increased by CYB5R3 overexpression (Fig. 6e). We further investigated the role of CYB5R3 in the ADP-ribosylation of PERK and IRE1α and found that it increased the ADP-ribosylation of PERK and IRE1α (Fig. 6f). Surprisingly, proteins immunoprecipitated with the anti-PERK or anti-IRE1α antibody bound to CYB5R3-Flag (Fig. 6f). Moreover, an immunoprecipitation assay using an anti-FLAG antibody showed that CYB5R3-Flag interacted with endogenous PERK and IRE1α in H1299 cells (Supplementary Fig. 5). These data suggest that CYB5R3 promotes the PARP16-mediated ADP-ribosylation of PERK and IRE1α to induce ER stress.

### CYB5R3 activates caspase-9 through oxidative stress

**Fig. 7:**
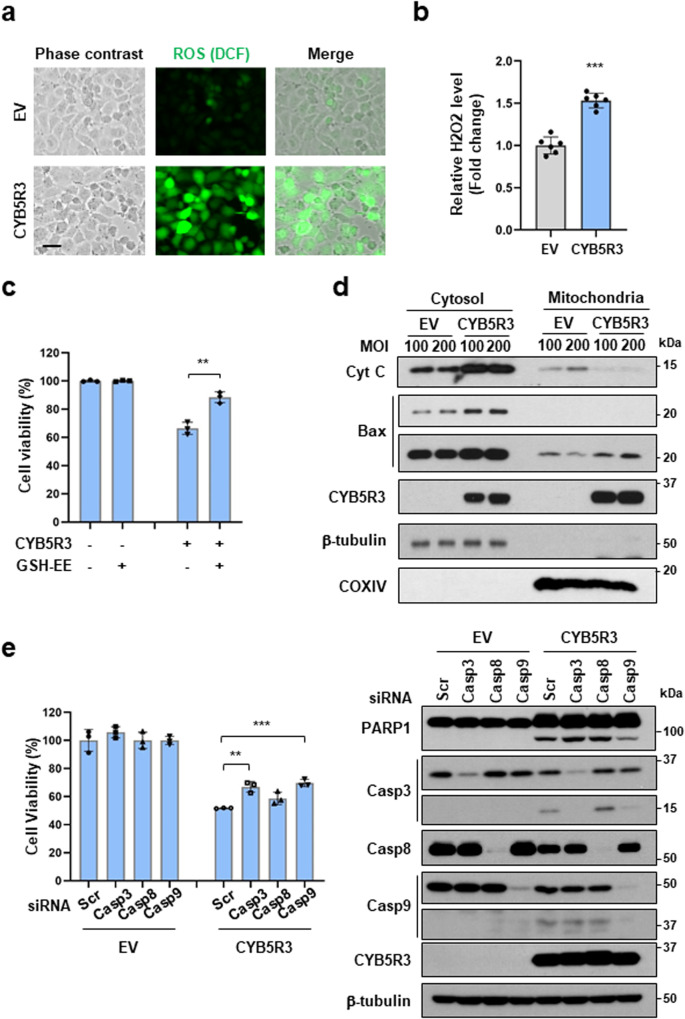
CYB5R3 mediates cell death via the intrinsic apoptotic pathway through ROS generation and caspase-9 activation. **a** DCF-DA staining in H1299 cells infected with CYB5R3 adenovirus for 24 h. Scale bar, 50 μm. **b** H_2_O_2_ levels in H1299 cells infected with CYB5R3 adenovirus for 24 h, as measured using the ROS-Glo H_2_O_2_ assay. **c** H1299 cells were pretreated with 1 mM GSH-EE for 1 h and infected with CYB5R3 for 72 h. Cell viability was determined using an SRB assay. **d** Subcellular fractionation was performed in H1299 cells infected with CYB5R3 for 24 h. Immunoblot analysis was performed using the indicated antibodies. **e** H1299 cells were transfected with the indicated siRNA and infected with CYB5R3 for 48 h. A cell viability assay (left panel) and immunoblot analysis (right panel) were performed. The values are presented as the mean ± SD of three independent experiments. **P < 0.01, ***P < 0.001.

Based on the increase in the level of oxidized GSH (GSSG) in CYB5R3-overexpressing cells (Fig. 5f), reactive oxygen species (ROS) generation was evaluated by DCF-DA staining. We observed ROS production in CYB5R3-overexpressing H1299 cells (Fig. 7a). Consistent with a previous report [33], we found that the H_2_O_2_ level was significantly increased in CYB5R3-transduced cells compared with EV-transduced cells (Fig. 7b). However, mitochondrial superoxide was not detected in CYB5R3-transduced cells (Supplementary Fig. 6). CYB5R3-induced H_2_O_2_ production was obviously decreased in NOX4-depleted cells (Supplementary Fig. 7). This result indicated that ROS production induced by CYB5R3 overexpression is associated with NOX4 expression in lung cancer cells. GSH-ethyl ester (GSH-EE), a cell-permeable derivative of GSH, prevented CYB5R3-induced cell death (Fig. 7c). To investigate whether CYB5R3 induces mitochondrial dysfunction, we isolated the mitochondrial and cytosolic fractions of CYB5R3-transduced cells. The level of Bax was increased in the mitochondrial fraction, whereas the level of cytochrome C was increased in the cytosolic fraction (Fig. 7d). Moreover, we found that knockdown of caspase-9 or caspase-3 decreased CYB5R3-induced cell death (Fig. 7e). These results suggested that CYB5R3 induces cell death cia the intrinsic apoptotic pathway through oxidative stress and caspase-9 activation.

## Discussion

**Fig. 8:**
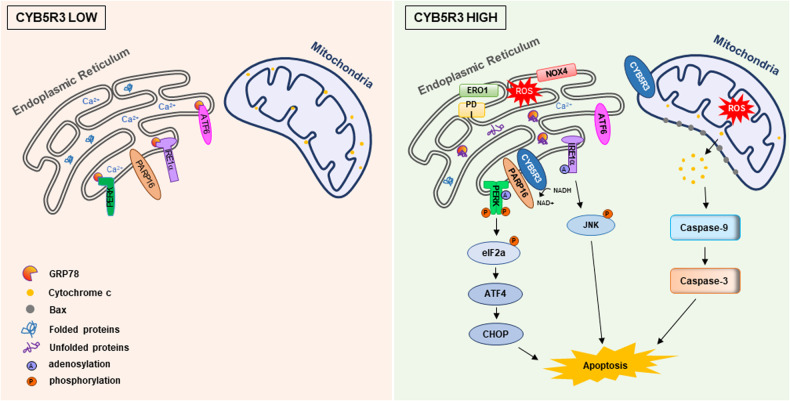
Mechanistic model for CYB5R3-induced lung cancer cell death. In lung cancer cells, CYB5R3 is downregulated, and CYB5R3 overexpression promotes cell death by inducing ER stress and ROS generation. CYB5R3 activates the PERK-ATF4-CHOP and IRE1α-JNK pathways through ADP-ribosylation mediated by PARP16. ROS generation induced by CYB5R3 activates caspase-9-dependent cell death.

Here, we showed that overexpression of CYB5R3 induces apoptosis in lung cancer cells in vitro and in vivo. In addition, the in vivo study in the CYB5R3 KO mouse model revealed the tumor-suppressive function of CYB5R3 in lung cancer. Transcriptome and metabolomic analyses indicated that CYB5R3 induces ER stress by activating PARP16-dependent ADP-ribosylation of PERK or IRE1α. Moreover, ROS generation induced by CYB5R3 activates the caspase-9-mediated intrinsic apoptotic pathway. Accumulated data on CYB5R3 have provided evidence that CYB5R3 inhibits lung cancer growth (Fig. 8).

Members of the CYB5R family have antioxidant properties and are expressed in several subcellular compartments, including the ER, mitochondrial outer membrane, and plasma membrane [38–41]. According to the Human Protein Atlas database, CYB5R1 is localized in mitochondria and the cytosol and is highly expressed in skeletal muscle. CYB5R2 localizes in the Golgi apparatus and nucleoplasm and is highly expressed in the testis. In CYB5R3-overexpressing H1299 cells, CYB5R3 is mainly localized in the ER and undergoes posttranslational modifications, leading to changes in its subcellular localization [42–44]. Recent studies have suggested that prenylated CYB5R3 translocates from mitochondria to the ER, while nonprenylated CYB5R3 localizes to mitochondria [43]. In particular, geranylgeranyl diphosphate synthase (GGPPS), which is involved in CYB5R3 prenylation, is overexpressed in lung adenocarcinoma [42]. In addition, ufmylation of CYB5R3, which negatively regulates its activity, occurs in the ER, and ufmylated CYB5R3 is degraded via CDK5RAP3 (CDK5 Regulatory Subunit Associated Protein 3)-mediated macro-ER-phagy [44].

NAD-dependent signaling events regulate numerous biological processes, including transcription, DNA repair, apoptosis, and metabolism. NAD^+^ is an important metabolite and substrate for enzymes such as poly(ADP-ribose) polymerases (PARPs) and sirtuins [34]. ADP-ribosylation is catalyzed by members of two different ADP-ribosyltransferase (ART) families, namely, the clostridial-toxin-like ADP-ribosyltransferase (ARTC) and diphtheria-toxin-like ART (ARTD) families [45]. The human ARTC family consists of four ecto-mono-ARTs: active mono-ARTs (ARTC1 and ARTC5) and inactive proteins (ARTC3 and ARTC4). The ARTD (also known as PARP) family contains 17 members: mono-ARTs (ARTD7-17), poly-ARTs (ARTD1-6), and the inactive protein ARTD13, which have distant subcellular localizations and protein substrates [46]. Recently, ARTC1 (ART1) and ARTD15 (PARP16) have been identified as ER-resident ARTs that mediate the mono-ADP-ribosylation of their substrates [36, 37]. Both ARTC1 and PARP16 are activated during ER stress and regulate the UPR in the ER. Mono-ADP-ribosylation of GRP78 by ARTC1 inactivates its chaperone activity and activates ER stress by dissociating it from its interactors, such as PERK, IRE1α, and ATF6 [36]. Mono-ADP-ribosylation of PERK and IRE1α by PARP16 increases their enzymatic activity and enhances ER stress responses [37]. Our data demonstrated that PARP16 plays a critical role in CYB5R3-induced lung cancer cell death by increasing ADP-ribosylation of PERK and IRE1α. Interestingly, we also found that CYB5R3 interacts with PERK and IRE1α. Therefore, further research is needed to determine whether the oxidation of PERK and IRE1α is required for their binding.

Mitochondria are the main source of ROS. Recent studies have suggested that the ER regulates redox homeostasis and retains relatively high ROS levels [47]. Oxidative protein folding occurs in the ER and generates ROS by catalyzing disulfide bond formation between protein disulfide isomerase (PDI) and ERO1 during protein folding [48–50]. ER protein oxidation and mitochondrial oxidative phosphorylation are sources of ROS generated during ER stress [51]. ROS generation by NADPH oxidase 4 (NOX4) in the ER membrane can induce apoptosis [52]. A recent study showed that CYB5R3 regulates NOX4-dependent H_2_O_2_ production and endothelial inflammation [33]. As expected, CYB5R3 decreased the GSH/GSSG ratio and increased NOX4-dependent H_2_O_2_ production, and treatment with cell-permeable GSH-EE attenuated apoptosis mediated by CYB5R3 overexpression in lung cancer cells. In addition, we observed that CYB5R3 significantly altered the abundances of metabolites, such as AMP and ADP, which induced the activation of the LKB1/AMPK pathway. Therefore, further studies are required to explore the role of CYB5R3 in tumor metabolism.

Although the role of CYB5R3 in cancer remains controversial, a previous study showed that CYB5R3 promotes cell colonization and metastasis formation in estrogen receptor-negative breast cancer [19]. However, another study demonstrated that CYB5R3 overexpression protected against chemically induced liver cancer in CYB5R3 transgenic mice [12]. In clear cell renal cell carcinoma, HADHA overexpression inhibits tumor growth by increasing the expression of CYB5R3 or ACAT1 [53]. The properties of CYB5R3 as a tumor suppressor in cancer cells can be exploited to develop anticancer drugs. CYB5R3-overexpressing vehicles, such as adenoviruses, lentiviruses, vaccinia viruses, and retroviruses, can be used to infect lung tumor cells. In addition, combination treatments with a CYB5R3-overexpressing vehicle and immune checkpoint inhibitors or cytokines can be developed to synergistically inhibit the growth of lung cancer.

In conclusion, CYB5R3 deficiency promotes tumorigenesis and lung metastasis in mouse models. CYB5R3 overexpression induces apoptosis in lung cancer cells via ER stress and ROS generation, suggesting approaches for the development of CYB5R3-based therapeutics for lung cancer.

### Supplementary information

Supplementary information

## References

[1] Bray F, Ferlay J, Soerjomataram I, Siegel RL, Torre LA, Jemal A (2018). Global cancer statistics 2018: GLOBOCAN estimates of incidence and mortality worldwide for 36 cancers in 185 countries. CA Cancer J. Clin..

[2] Osmani L, Askin F, Gabrielson E, Li QK (2018). Current WHO guidelines and the critical role of immunohistochemical markers in the subclassification of non-small cell lung carcinoma (NSCLC): Moving from targeted therapy to immunotherapy. Semin. Cancer Biol..

[3] Pikor LA, Ramnarine VR, Lam S, Lam WL (2013). Genetic alterations defining NSCLC subtypes and their therapeutic implications. Lung Cancer.

[4] George J, Lim JS, Jang SJ, Cun Y, Ozretic L, Kong G (2015). Comprehensive genomic profiles of small cell lung cancer. Nature.

[5] Lockwood WW, Wilson IM, Coe BP, Chari R, Pikor LA, Thu KL (2012). Divergent genomic and epigenomic landscapes of lung cancer subtypes underscore the selection of different oncogenic pathways during tumor development. PLoS One.

[6] de Cabo R, Siendones E, Minor R, Navas P (2009). CYB5R3: a key player in aerobic metabolism and aging?. Aging.

[7] Jaffe ER (1981). Methemoglobin pathophysiology. Prog. Clin. Biol. Res..

[8] Villalba JM, Navarro F, Gomez-Diaz C, Arroyo A, Bello RI, Navas P (1997). Role of cytochrome b5 reductase on the antioxidant function of coenzyme Q in the plasma membrane. Mol. Aspects Med..

[9] Straub AC, Lohman AW, Billaud M, Johnstone SR, Dwyer ST, Lee MY (2012). Endothelial cell expression of haemoglobin alpha regulates nitric oxide signalling. Nature.

[10] Rahaman MM, Nguyen AT, Miller MP, Hahn SA, Sparacino-Watkins C, Jobbagy S (2017). Cytochrome b5 reductase 3 modulates soluble guanylate cyclase redox state and cGMP signaling. Circ. Res..

[11] Oshino N, Imai Y, Sato R (1971). A function of cytochrome b5 in fatty acid desaturation by rat liver microsomes. J. Biochem..

[12] Martin-Montalvo A, Sun Y, Diaz-Ruiz A, Ali A, Gutierrez V, Palacios HH (2016). Cytochrome b5 reductase and the control of lipid metabolism and healthspan. NPJ Aging Mech. Dis..

[13] Reddy VV, Kupfer D, Caspi E (1977). Mechanism of C-5 double bond introduction in the biosynthesis of cholesterol by rat liver microsomes. J. Biol. Chem..

[14] Hildebrandt A, Estabrook RW (1971). Evidence for the participation of cytochrome b 5 in hepatic microsomal mixed-function oxidation reactions. Arch. Biochem. Biophys..

[15] Sacco JC, Trepanier LA (2010). Cytochrome b5 and NADH cytochrome b5 reductase: genotype-phenotype correlations for hydroxylamine reduction. Pharmacogenet Genomics.

[16] Durgin, B.G., Hahn, S.A., Schmidt, H.M., Miller, M.P., Hafeez, N., Mathar, I. et al. (2019). Loss of smooth muscle CYB5R3 amplifies angiotensin II-induced hypertension by increasing sGC heme oxidation. *JCI Insight* **4**, 10.1172/jci.insight.129183 (2019). 10.1172/jci.insight.129183 PMC6795404 31487266

[17] Siendones E, SantaCruz-Calvo S, Martin-Montalvo A, Cascajo MV, Ariza J, Lopez-Lluch G (2014). Membrane-bound CYB5R3 is a common effector of nutritional and oxidative stress response through FOXO3a and Nrf2. Antioxid Redox Signal.

[18] Fan J, Du W, Kim-Muller JY, Son J, Kuo T, Larrea D (2020). Cyb5r3 links FoxO1-dependent mitochondrial dysfunction with beta-cell failure. Mol. Metab..

[19] Lund RR, Leth-Larsen R, Caterino TD, Terp MG, Nissen J, Laenkholm AV (2015). NADH-Cytochrome b5 reductase 3 promotes colonization and metastasis formation and is a prognostic marker of disease-free and overall survival in estrogen receptor-negative breast cancer. Mol. Cell Proteomics.

[20] Szegezdi E, Logue SE, Gorman AM, Samali A (2006). Mediators of endoplasmic reticulum stress-induced apoptosis. EMBO Rep..

[21] Tabas I, Ron D (2011). Integrating the mechanisms of apoptosis induced by endoplasmic reticulum stress. Nat. Cell Biol..

[22] Hetz C (2012). The unfolded protein response: controlling cell fate decisions under ER stress and beyond. Nat. Rev. Mol. Cell Biol..

[23] Schroder M, Kaufman RJ (2005). The mammalian unfolded protein response. Annu. Rev. Biochem..

[24] Urano F, Wang X, Bertolotti A, Zhang Y, Chung P, Harding HP (2000). Coupling of stress in the ER to activation of JNK protein kinases by transmembrane protein kinase IRE1. Science.

[25] Nishitoh H, Matsuzawa A, Tobiume K, Saegusa K, Takeda K, Inoue K (2002). ASK1 is essential for endoplasmic reticulum stress-induced neuronal cell death triggered by expanded polyglutamine repeats. Genes Dev..

[26] Miller YE, Dwyer-Nield LD, Keith RL, Le M, Franklin WA, Malkinson AM (2003). Induction of a high incidence of lung tumors in C57BL/6 mice with multiple ethyl carbamate injections. Cancer Lett..

[27] Jao LE, Wente SR, Chen W (2013). Efficient multiplex biallelic zebrafish genome editing using a CRISPR nuclease system. Proc. Natl Acad. Sci. USA.

[28] Im JY, Lee KW, Won KJ, Kim BK, Ban HS, Yoon SH (2016). DNA damage-induced apoptosis suppressor (DDIAS), a novel target of NFATc1, is associated with cisplatin resistance in lung cancer. Biochim. Biophys. Acta.

[29] Vichai V, Kirtikara K (2006). Sulforhodamine B colorimetric assay for cytotoxicity screening. Nat. Protoc..

[30] Kurashige T, Shimamura M, Hamada K, Matsuse M, Mitsutake N, Nagayama Y (2023). Characterization of metabolic reprogramming by metabolomics in the oncocytic thyroid cancer cell line XTC.UC1. Sci. Rep..

[31] Sanjana NE, Shalem O, Zhang F (2014). Improved vectors and genome-wide libraries for CRISPR screening. Nat. Methods.

[32] Oyadomari S, Mori M (2004). Roles of CHOP/GADD153 in endoplasmic reticulum stress. Cell Death Differ..

[33] Yuan S, Hahn SA, Miller MP, Sanker S, Calderon MJ, Sullivan M (2021). Cooperation between CYB5R3 and NOX4 via coenzyme Q mitigates endothelial inflammation. Redox. Biol..

[34] Chiarugi A, Dolle C, Felici R, Ziegler M (2012). The NAD metabolome–a key determinant of cancer cell biology. Nat. Rev. Cancer.

[35] Houtkooper RH, Pirinen E, Auwerx J (2012). Sirtuins as regulators of metabolism and healthspan. Nat. Rev. Mol. Cell Biol..

[36] Fabrizio G, Di Paola S, Stilla A, Giannotta M, Ruggiero C, Menzel S (2015). ARTC1-mediated ADP-ribosylation of GRP78/BiP: a new player in endoplasmic-reticulum stress responses. Cell Mol. Life Sci..

[37] Jwa M, Chang P (2012). PARP16 is a tail-anchored endoplasmic reticulum protein required for the PERK- and IRE1alpha-mediated unfolded protein response. Nat. Cell Biol..

[38] De Cabo R, Cabello R, Rios M, Lopez-Lluch G, Ingram DK, Lane MA (2004). Calorie restriction attenuates age-related alterations in the plasma membrane antioxidant system in rat liver. Exp. Gerontol..

[39] Navarro F, Villalba JM, Crane FL, Mackellar WC, Navas P (1995). A phospholipid-dependent NADH-coenzyme Q reductase from liver plasma membrane. Biochem. Biophys. Res. Commun..

[40] Zhu H, Larade K, Jackson TA, Xie J, Ladoux A, Acker H (2004). NCB5OR is a novel soluble NAD(P)H reductase localized in the endoplasmic reticulum. J. Biol. Chem..

[41] Zhao X, Leon IR, Bak S, Mogensen M, Wrzesinski K, Hojlund K (2011). Phosphoproteome analysis of functional mitochondria isolated from resting human muscle reveals extensive phosphorylation of inner membrane protein complexes and enzymes. Mol. Cell Proteomics.

[42] Wang X, Xu W, Zhan P, Xu T, Jin J, Miu Y (2018). Overexpression of geranylgeranyl diphosphate synthase contributes to tumour metastasis and correlates with poor prognosis of lung adenocarcinoma. J. Cell Mol. Med..

[43] Wei L, Zheng YY, Sun J, Wang P, Tao T, Li Y (2020). GGPP depletion initiates metaflammation through disequilibrating CYB5R3-dependent eicosanoid metabolism. J. Biol. Chem..

[44] Ishimura R, El-Gowily AH, Noshiro D, Komatsu-Hirota S, Ono Y, Shindo M (2022). The UFM1 system regulates ER-phagy through the ufmylation of CYB5R3. Nat. Commun..

[45] Di Girolamo M, Fabrizio G (2019). Overview of the mammalian ADP-ribosyl-transferases clostridia toxin-like (ARTCs) family. Biochem. Pharmacol..

[46] Vyas S, Chesarone-Cataldo M, Todorova T, Huang YH, Chang P (2013). A systematic analysis of the PARP protein family identifies new functions critical for cell physiology. Nat. Commun..

[47] Bhattarai KR, Riaz TA, Kim HR, Chae HJ (2021). The aftermath of the interplay between the endoplasmic reticulum stress response and redox signaling. Exp. Mol. Med..

[48] Enyedi B, Varnai P, Geiszt M (2010). Redox state of the endoplasmic reticulum is controlled by Ero1L-alpha and intraluminal calcium. Antioxid. Redox. Signal.

[49] Araki K, Iemura S, Kamiya Y, Ron D, Kato K, Natsume T (2013). Ero1-alpha and PDIs constitute a hierarchical electron transfer network of endoplasmic reticulum oxidoreductases. J. Cell Biol..

[50] Tu BP, Weissman JS (2004). Oxidative protein folding in eukaryotes: mechanisms and consequences. J. Cell Biol..

[51] Bhandary B, Marahatta A, Kim HR, Chae HJ (2012). An involvement of oxidative stress in endoplasmic reticulum stress and its associated diseases. Int. J. Mol. Sci..

[52] Sciarretta S, Zhai P, Shao D, Zablocki D, Nagarajan N, Terada LS (2013). Activation of NADPH oxidase 4 in the endoplasmic reticulum promotes cardiomyocyte autophagy and survival during energy stress through the protein kinase RNA-activated-like endoplasmic reticulum kinase/eukaryotic initiation factor 2alpha/activating transcription factor 4 pathway. Circ. Res..

[53] Liu S, Liu X, Wu F, Zhang X, Zhang H, Gao D (2019). HADHA overexpression disrupts lipid metabolism and inhibits tumor growth in clear cell renal cell carcinoma. Exp. Cell Res..

