# Supplementary material for: CYB5R3 functions as a tumor suppressor by inducing ER stress-mediated apoptosis in lung cancer cells via the PERK-ATF4 and IRE1α-JNK pathways
Source: Exp Mol Med. 2024 Jan 22;56(1):235–49. doi: 10.1038/s12276-024-01155-9 (PMC10834511; doi:10.1038/s12276-024-01155-9)
Supplement: Supplementary file 1 — Supplementary information [file 12276_2024_1155_MOESM1_ESM.pdf]

Supplementary information

MATERIAL AND METHODS

Materials

MitoTracker (M7512), MitoSOX Red (M36008) and NOX4 siRNA (HSS121312) were purchased from Thermo Scientific. (Rockford, IL, USA). Anti-NOX4 (ab109225) were obtained from Abcam (Cambridge, UK). Anti-Flag-M2 affinity agarose beads (A2220) were obtained from Sigma-Aldrich (St. Louis, MO, USA). Anti-CYB5R1 (sc-160051) or anti-CYB5R2 (sc-161509) were obtained from Santa Cruz Biotechnology (Dallas, TX, USA).

Mitochondrial superoxide staining

MitoSOX Red was used to measure mitochondrial superoxide according to the manufacturer's instructions. Briefly, H1299 cells infected with EV or CYB5R3 for 24 h were stained with MitoSOX Red (1 μM) for 30 min and washed three times with HBSS. Images were acquired using CKX53 fluorescent microscope (Olympus, Tokyo, Japan).

Cell culture

The Lewis lung cancer (LLC) cells were purchased from ATCC (Manassas, VA, USA). LLC cells were cultured in Dulbecco's modified Eagle's medium (DMEM) supplemented with 10% fetal bovine serum (FBS), penicillin/streptomycin (Invitrogen, Carlsbad, CA, USA).

Animal experiments

All mouse experiments were conducted in accordance with a protocol approved by the Institutional Animal Care and Use Committee. C57BL/6 mice were purchased from the ORIENT BIO (Gyeonggi-do, Korea). For all tissues including brain, heart, lung, liver, stomach,

spleen, kidney, testis and ovary from control mice were placed in cryogenic vials and frozen immediately in liquid nitrogen. All tissues were stored at -80°C until the analysis.

For isolation of mouse lung fibroblast, C57BL/6 mice were sacrificed and the lungs were removed and digested, and the resulting lung cells were plated and cultured as described previously[1].

For LLC metastasis mouse models, CYB5R3$^{+/+}$ or CYB5R3$^{-/-}$ mice were used at 7-9 weeks of age. $5x10^5$ LLC cells were inoculated into subcutaneous dorsal area in a volume of 100 μl. The size of primary tumors was measured regularly every 3 days using calipers and calculated according to the formula [(lengthXwidth2) /2], where length represents the larger tumor diameter and width represents the smaller tumor diameter. Primary tumors were surgically removed when they reached approximately 500 mm^3 in tumor volume. Metastatic tumor formation in the lungs were sacrificed after 4 weeks. Mice were sacrificed every week after LLC cell injection and tumor tissues were harvested for further analyses. Lungs were fixed in 10% buffered formalin and embedded in paraffin. Histological sections (5 μm) were stained with hematoxylin and eosin and analyzed in an Olympus microscope.

Supplementary Fig. 1. Downregulation of CYB5R3 in human tumors. a CYB5R3 expression profile across all tumor samples and corresponding normal tissues in the TCGA database. **b** Representative images and scores of IHC staining showing CYB5R3 expression in human lung cancer tissue array. IHC scores defined 0-3+ according to staining intensity. Scale bar, 100 μm.

Supplementary Fig. 2. Expression of CYB5R1 and CYB5R2 in human lung cancer. a CYB5R1 expression level in normal lung tissue and lung cancer patients. **b** CYB5R2 expression level in normal lung tissue and lung cancer patients. The expression data was

collected in TCGA cohort cBioPortal (http://cbioportal.org) and then visualized by ggplot2 (version 3.3.6). All statistical analyses were performed using R software. **c** Protein levels of CYB5R1 or CYB5R2 in 2 human lung fibroblasts and 8 lung cancer cell lines.

Supplementary Fig. 3. Validation of CYB5R3 knockout mouse models. a The expression levels of CYB5R3 in mouse tissues. SE (short exposure), LE (long exposure). **b** Comparison of CYB5R3 expression between mouse normal lung fibroblast and LLC cells. **c** Schematic presentation of CYB5R3 KO mice generation. **d** Body weight in CYB5R3$^{+/+}$ or CYB5R3$^{-/-}$ mice (n=24 per group). **e** Immunoblot analysis of the indicated mouse tissues in CYB5R3$^{+/+}$, CYB5R3$^{+/-}$ or CYB5R3$^{-/-}$ mice (n=3 per group). **f** Tumor burden metastasized to lung after injection of LLC into tail vein in CYB5R3$^{+/+}$ or CYB5R3$^{-/-}$ mice (n=3 per group). **g** H&E staining of lung tissues in CYB5R3$^{+/+}$ or CYB5R3$^{-/-}$ mice at the indicated times after injection of LLC into tail vein (n=3 per group). Scale bar, 100 μm.

Supplementary Fig. 4. Mitochondrial localization of CYB5R3. Immunofluorescence staining of Flag (green) and Mito tracker (red) was carried out in H1299 cells infected with EV or CYB5R3 for 24 h. DAPI (blue) was used for nuclei staining. Scale bar, 10 μm.

Supplementary Fig. 5. CYB5R3 interacts with PERK and IRE1α. The immunoprecipitation was performed with anti-Flag-M2 affinity agarose in H1299 cells infected with EV or CYB5R3 for 24 h. Immunoprecipitates were performed by immunoblot analysis using the indicated antibodies.

Supplementary Fig. 6. Mitochondrial superoxide levels in CYB5R3 overexpressed cells. Mitochondrial superoxide was measured using MitoSOX Red in H1299 cells infected with EV

or CYB5R3 for 24 h. Scale bar, 50 μm.

Supplementary Fig. 7. NOX4 regulates CYB5R3-induced H_2O_2 production. a H_2O_2 was measured using ROS-Glo H_2O_2 assay in H1299 cells infected with EV or CYB5R3 for 24 h following transfection with siRNA against NOX4 (siNOX4) or scramble (siScr). n=5; * indicates $p < 0.05$ between indicated groups with two-way ANOVA with multiple comparison test. **b** H1299 cells were co-transfected CYB5R3 with siNOX4 or siScr. Protein level of NOX4 and CYB5R3 was measured by immunoblot analysis at 72 h after transfection.

REFERENCE

1. Seluanov, A., Vaidya, A. & Gorbunova, V. Establishing primary adult fibroblast cultures from rodents. *J Vis Exp* (2010).

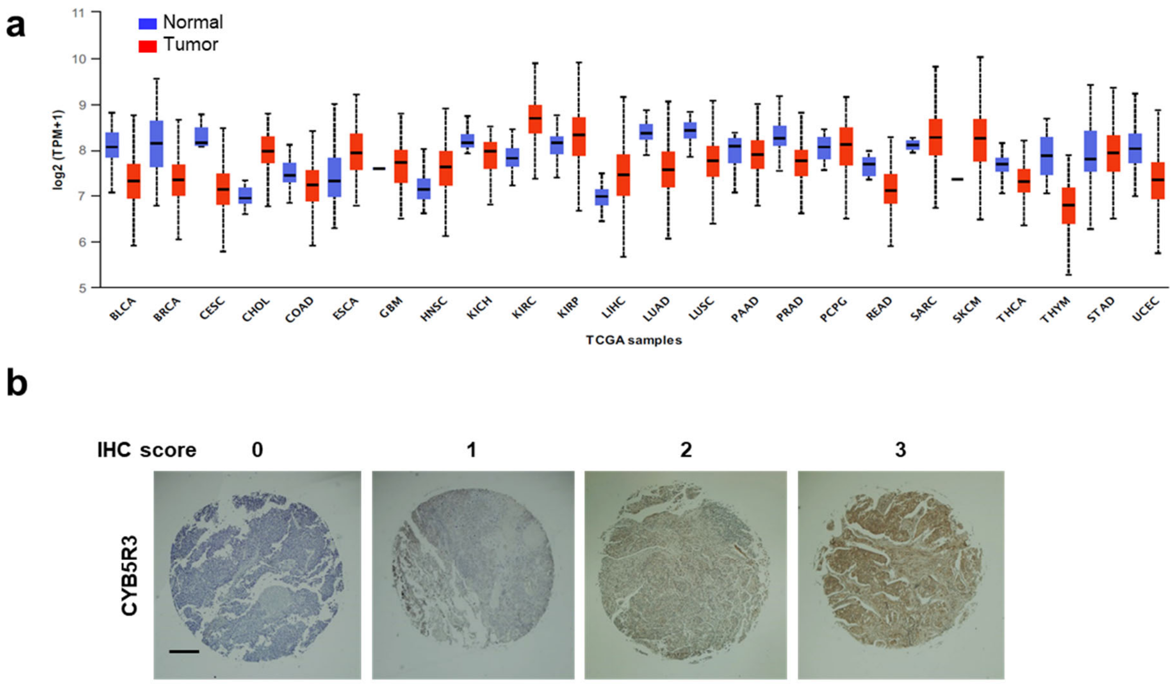

Supplementary Fig. 1

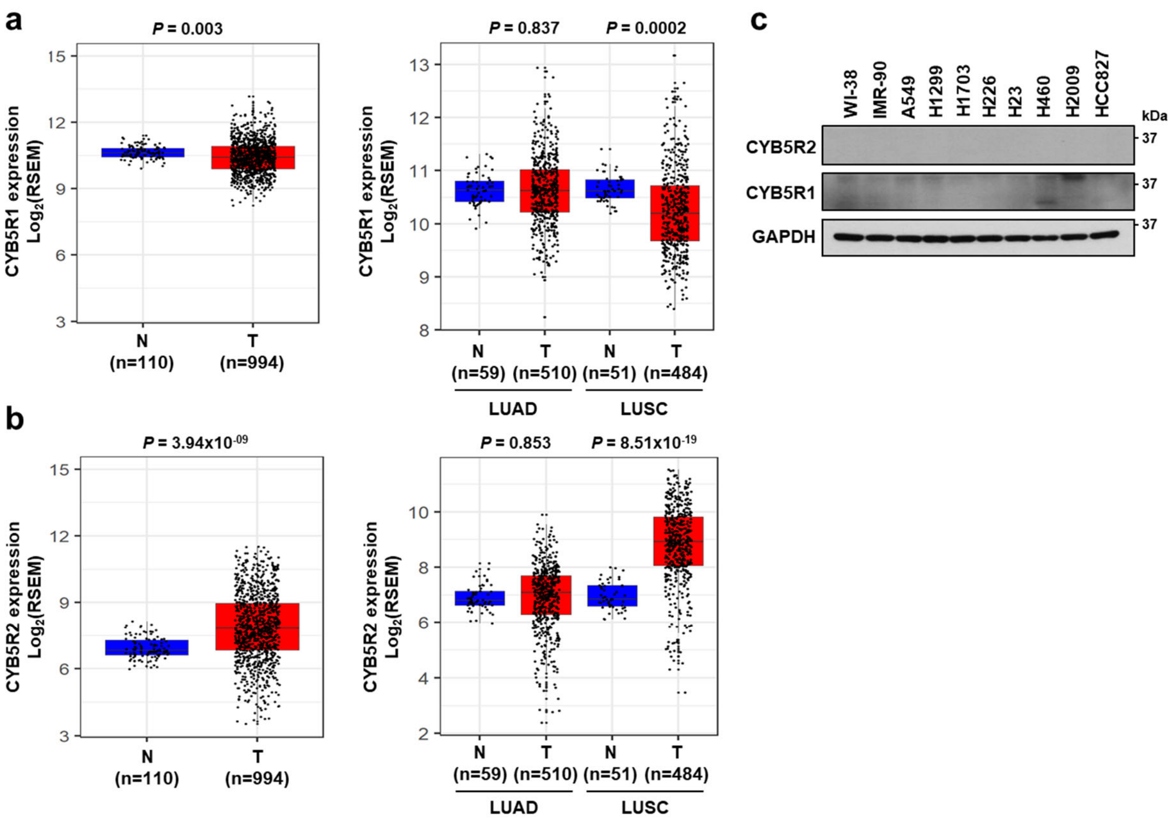

Supplementary Fig. 2

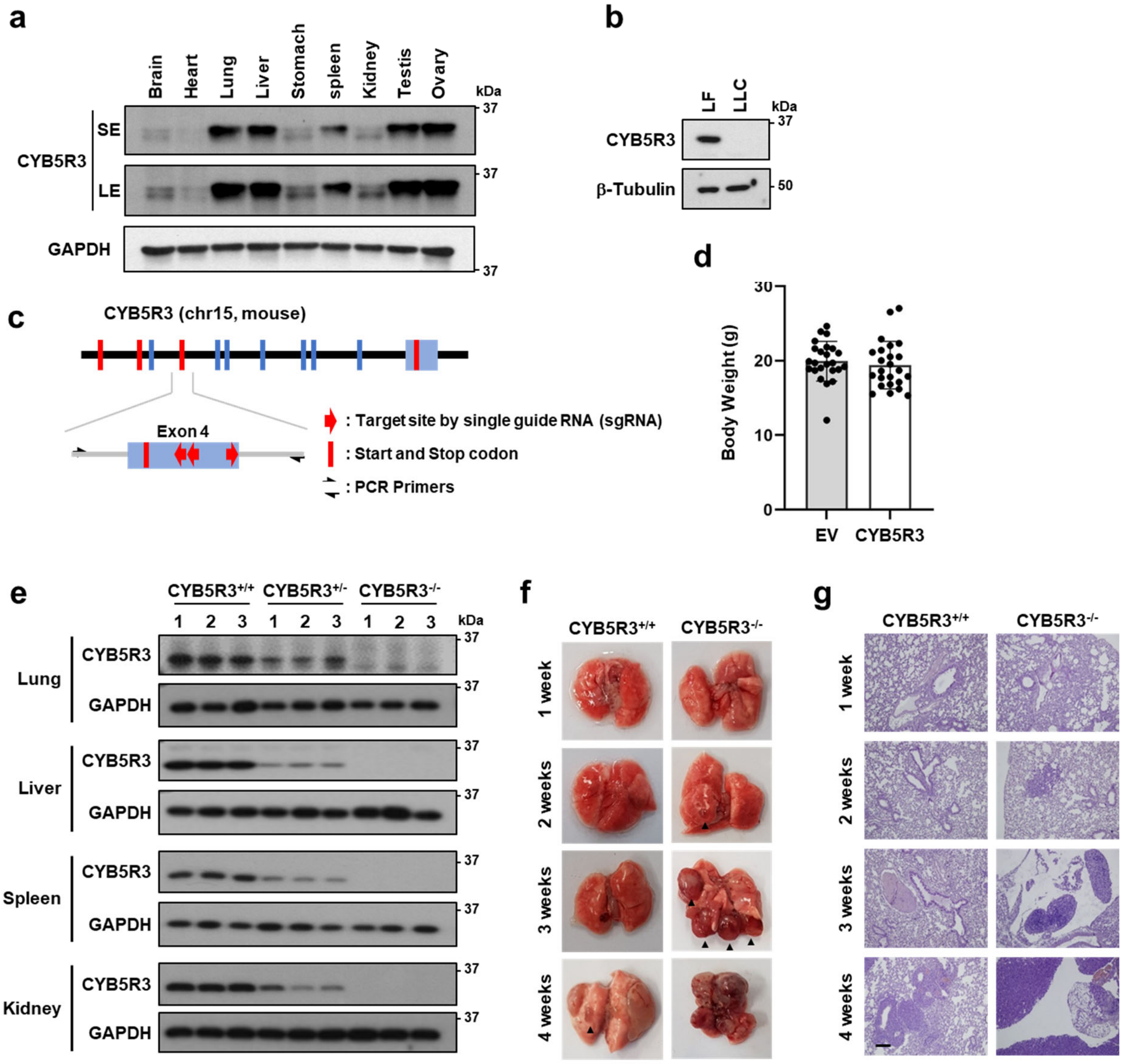

Supplementary Fig. 3

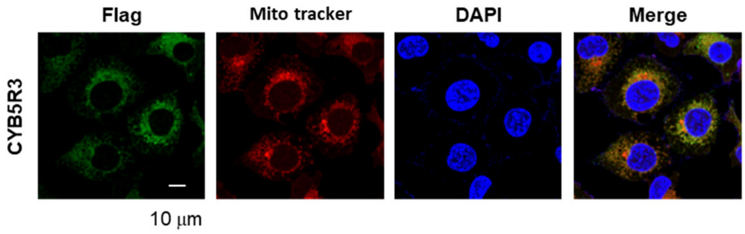

Supplementary Fig. 4

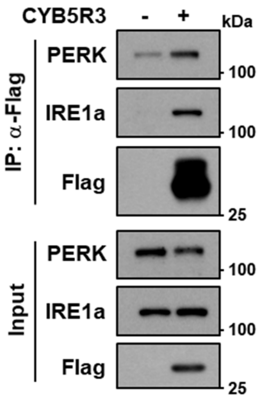

Supplementary Fig. 5

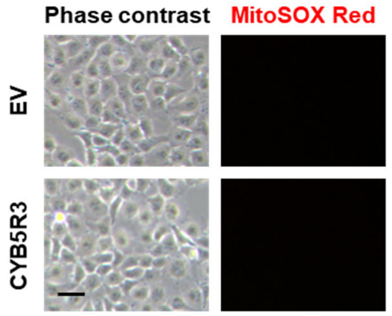

Supplementary Fig. 6

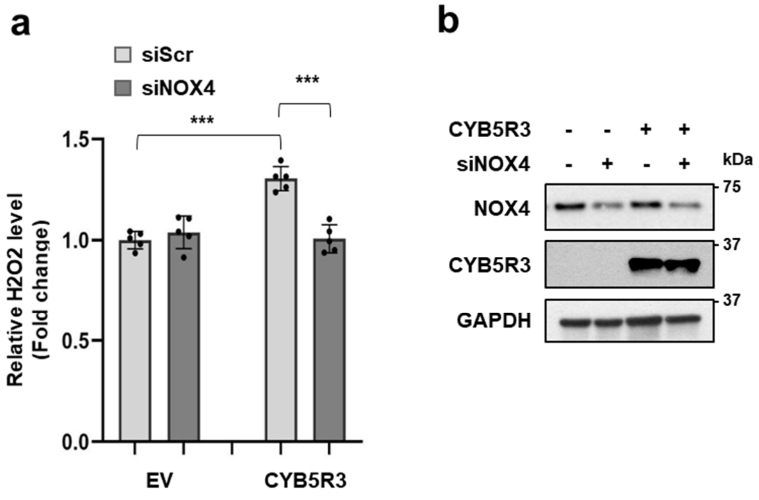

Supplementary Fig. 7

Supplementary Table 1. Hematology of WT and CYB5R3 KO mice

	WT (n=3)			CYB5R3 KO (n=3)			*p*-value
	1	2	3	1	2	3	
WBC (White blood cell) (1×10^3/ ul)	6.07	7.14	7.84	5.13	3.34	3.72	*0.017
Lymphocytes (1×10^3/ ul)	5.37	5.02	5.84	4.56	2.95	2.77	*0.032
Monocytes (1×10^3/ ul)	0.27	0.22	0.14	0.12	0.13	0.07	0.070
RBC (Red blood cell) (1×10^6/ ul)	9.51	8.98	8.53	8.81	9.18	8.18	0.524
Hemoglobin (g/dl)	14.80	13.40	13.20	13.30	13.60	12.60	0.339
Hematocrit (%)	48.70	45.00	42.90	44.50	46.50	39.90	0.503
Platelet (1×10^3/ ul)	509	449	505	1023	50	238	0.874

g/dl is grams per deciliter. *P* values are unpaired 2-tailed t test. * indicates $P<0.05$ between WT and CYB5R3 KO with unpaired parametric *t*-test

Table 2. Absolute concentration of the intracellular metabolite level in H1299 cells infected with EV (AdEV-24), CYB5R3 (AdR3-24) for 24 h or EV (AdEV-36), CYB5R3 (AdR3-36) for 36 h.

ID	Compound name	Pathway Label	ubChem CI	HMDB ID	AdEV-24			AdR3-24			AdEV-36			AdR3-36		
					1	2	3	1	2	3	1	2	3	1	2	3
A_0001	NAD^+	NAD+	5893	HMDB0000902	3,366	3,095	3,494	4,562	4,577	4,195	3,770	3,775	3,763	5,123	4,855	5,125
A_0002	cAMP	cAMP	6076	HMDB0000058	78	230	169	115	109	133	132	42	47	21	22	20
A_0003	cGMP	cGMP	24316	HMDB0001314	N.D.	N.D.	N.D.	N.D.	N.D.	N.D.	0.11	N.D.	N.D.	N.D.	N.D.	0.15
A_0004	NADH	NADH	439153	HMDB0001487	239	218	246	246	234	223	231	220	205	201	176	201
A_0005	Xanthine	Xanthine	1188	HMDB0000292	29	24	25	45	38	38	17	20	20	26	27	25
A_0006	ADP-ribose	ADP-Rib	445794	HMDB0001178	3.8	3.9	2.6	5.2	5.5	5.4	4.5	3.8	4.7	5.2	5.6	4.6
A_0007	Mevalonic acid	Mevalonic acid	134965	HMDB0000227	N.D.	N.D.	N.D.	N.D.	N.D.	N.D.	N.D.	N.D.	N.D.	N.D.	N.D.	N.D.
A_0008	UDP-glucose	UDP-Glc	8629	HMDB0000286	3,205	2,975	3,014	3,240	3,305	2,691	5,464	5,605	5,163	7,284	7,694	7,286
A_0009	Uric acid	Uric acid	1175	HMDB0000289	N.D.	N.D.	N.D.	N.D.	N.D.	N.D.	2.0	3.5	1.2	N.D.	1.2	0.3
A_0010	$NADP^+$	NADP+	5886	HMDB0000217	162	134	136	191	228	158	211	214	200	281	272	254
A_0011	IMP	IMP	8582	HMDB0000175	67	53	53	72	74	73	11	11	11	19	18	17
A_0012	Sedoheptulose 7-phosphate	S7P	165007	HMDB0001068	N.D.	N.D.	N.D.	N.D.	N.D.	N.D.	N.D.	N.D.	N.D.	N.D.	N.D.	N.D.
A_0013	Glucose 6-phosphate	G6P	5958	HMDB0001401	51	42	41	34	32	15	98	113	108	66	63	59
A_0014	Fructose 6-phosphate	F6P	603	HMDB0000124	5.5	8.2	5.3	6.8	2.3	4.0	16	16	17	7.0	7.8	7.4
A_0015	Fructose 1-phosphate	D-F1P	439394	HMDB0001076	93	100	82	80	44	38	168	143	153	158	173	176
A_0016	Galactose 1-phosphate	Gal1P	123912	HMDB0000645	59	57	54	42	51	25	39	59	58	82	73	83
A_0017	Glucose 1-phosphate	G1P	65533	HMDB0001586	24	13	15	34	23	26	51	31	37	30	54	48
A_0018	Acetoacetyl CoA	AAcCoA	92153	HMDB0001484	N.D.	N.D.	N.D.	N.D.	N.D.	N.D.	N.D.	N.D.	N.D.	N.D.	N.D.	N.D.
A_0019	Acetyl CoA	AcCoA	444493	HMDB0001206	1.7	1.5	3.4	2.8	4.2	5.5	1.3	2.3	1.0	6.3	1.6	1.4
A_0020	Folic acid	Folic acid	6037	HMDB0000121	1.4	1.5	1.6	2.3	2.3	2.3	0.9	1.0	1.0	1.5	1.4	1.3
A_0021	Ribose 5-phosphate	R5P	439167	HMDB0001548	63	64	45	69	58	37	40	27	32	20	32	26
A_0022	CoA	CoA	87642	HMDB0001423	170	143	156	191	193	178	116	110	92	105	116	117
A_0023	Ribose 1-phosphate	R1P	439236	HMDB0001489	32	27	36	29	38	37	58	52	50	34	54	41
A_0024	Ribulose 5-phosphate	Ru5P	439184	HMDB0000618	76	54	89	70	64	33	21	17	13	19	23	20
A_0025	Xylulose 5-phosphate	X5P	439190	HMDB0000868	N.D.	N.D.	N.D.	N.D.	N.D.	N.D.	N.D.	N.D.	N.D.	N.D.	N.D.	N.D.
A_0026	Erythrose 4-phosphate	E4P	122357	HMDB0001321	N.D.	N.D.	N.D.	N.D.	N.D.	N.D.	N.D.	N.D.	N.D.	N.D.	N.D.	N.D.
A_0027	HMG CoA	HMG-CoA	445127	HMDB0001375	43	42	48	56	57	53	24	24	20	48	50	50
A_0028	Glyceraldehyde 3-phosphate	Glyceraldehyde 3-phosphate	729	HMDB0001112	N.D.	N.D.	N.D.	N.D.	N.D.	N.D.	N.D.	N.D.	N.D.	N.D.	N.D.	N.D.
A_0029	NADPH	NADPH	5884	HMDB0000221	110	112	129	135	112	130	93	95	97	90	91	116
A_0030	Malonyl CoA	Malonyl-CoA	644066	HMDB0001175	6.7	6.1	7.2	8.6	9.2	8.9	4.2	4.2	2.7	6.9	6.4	5.1
A_0031	Phosphocreatine	Phosphocreatine	9548602	HMDB0001511	2,379	2,148	2,394	2,202	2,240	1,941	3,604	3,416	3,452	3,826	3,858	3,780
A_0032	XMP	XMP	73323	HMDB0001554	9.3	6.6	7.6	8.7	9.1	8.2	11	4.9	13	6.5	6.3	5.6
A_0033	Dihydroxyacetone phosphate	DHAP	668	HMDB0001473	35	N.D.	N.D.	N.D.	N.D.	N.D.	216	141	109	45	38	35
A_0034	Adenylosuccinic acid	Succinyl AMP	447145	HMDB0000536	74	79	77	120	117	113	4.2	3.6	4.7	6.2	6.5	6.2
A_0035	Fructose 1,6-diphosphate	F1,6P	172313	HMDB0001058	1,369	956	1,232	684	839	424	3,354	3,325	3,170	2,272	2,624	2,379
A_0036	6-Phosphogluconic acid	6-PG	91493	HMDB0001316	37	26	25	38	34	34	28	32	32	36	32	32
A_0037	*N*-Carbamoylaspartic acid	Carbamoyl-Asp	93072	HMDB0000828	172	162	169	219	223	231	42	39	42	48	42	43
A_0038	PRPP	PRPP	7339	HMDB0000280	4,333	3,048	4,474	3,523	4,008	2,580	3,789	3,309	3,728	3,274	3,602	3,283
A_0039	2-Phosphoglyceric acid	2-PG	439278	HMDB0003391	39	40	65	54	46	52	26	25	27	34	44	38
A_0040	2,3-Diphosphoglyceric acid	Diphosphoglycerate	186004	HMDB0001294	68	67	82	47	44	43	60	58	66	73	64	74
A_0041	3-Phosphoglyceric acid	3-PG	439183	HMDB0000807	363	354	554	473	373	419	232	202	264	308	386	355
A_0042	Phosphoenolpyruvic acid	PEP	1005	HMDB0000263	134	147	212	168	149	159	82	81	92	100	120	115
A_0043	GMP	GMP	6804	HMDB0001397	43	52	35	168	160	169	20	22	17	49	54	44
A_0044	AMP	AMP	6083	HMDB0000045	222	332	260	1,207	1,154	1,262	80	65	53	148	144	103
A_0045	2-Oxoisovaleric acid	2-KIV	49	HMDB0000019	69	68	67	105	110	101	46	48	48	64	72	63
A_0046	GDP	GDP	8977	HMDB0001201	201	300	256	723	736	882	119	123	112	209	227	182
A_0047	Lactic acid	Lactic acid	612	HMDB0000190,HMDB0001311	87,726	83,633	82,696	131,003	133,314	127,054	54,481	54,374	55,274	81,561	82,926	80,723
A_0048	ADP	ADP	6022	HMDB0001341	1,165	1,543	1,369	3,996	3,679	4,582	565	506	455	1,005	1,026	843
A_0049	GTP	GTP	6830	HMDB0001273	4,500	4,294	3,886	4,754	4,924	4,372	5,584	5,785	5,492	7,151	7,176	7,080
A_0050	Glyoxylic acid	Glyoxylic acid	760	HMDB0000119	N.D.	N.D.	N.D.	N.D.	N.D.	N.D.	N.D.	N.D.	N.D.	N.D.	N.D.	N.D.
A_0051	ATP	ATP	5957	HMDB0000538	17,355	16,116	15,571	19,731	19,686	18,955	18,048	18,843	18,047	23,418	22,795	22,890
A_0052	Glycerol 3-phosphate	Glycerol 3-phosphate	439162	HMDB0000126	199	216	237	302	335	320	277	279	274	304	317	255
A_0053	Glycolic acid	Glycolic acid	757	HMDB0000115	N.D.	N.D.	N.D.	N.D.	N.D.	N.D.	N.D.	N.D.	N.D.	N.D.	N.D.	N.D.
A_0054	Pyruvic acid	Pyruvic acid	1060	HMDB0000243	418	398	361	353	502	478	176	159	171	263	212	153
A_0055	*N*-Acetylglutamic acid	N-AcGlu	70914	HMDB0001138	190	187	192	252	237	196	145	146	149	171	176	161
A_0056	2-Hydroxyglutaric acid	2-Hydroxyglutaric acid	43	HMDB0000606,HMDB0000694	93	97	88	133	141	158	65	63	53	97	93	84
A_0057	Carbamoylphosphate	Carbamoyl-P	278	HMDB0001096	N.D.	N.D.	N.D.	N.D.	N.D.	N.D.	N.D.	N.D.	N.D.	N.D.	N.D.	N.D.
A_0058	Succinic acid	Succinic acid	1110	HMDB0000254	618	613	565	2,715	2,655	2,569	113	120	150	407	410	360
A_0059	Malic acid	Malic acid	525	HMDB0000156,HMDB0000744	6,657	6,145	6,196	11,225	10,825	10,799	1,945	1,681	1,803	2,289	2,025	2,136
A_0060	2-Oxoglutaric acid	2-OG	51	HMDB0000208	967	991	824	682	586	720	225	164	0.8	195	177	120
A_0061	Fumaric acid	Fumaric acid	444972	HMDB0000134	1,078	1,147	1,083	2,018	1,903	1,911	276	218	258	260	249	244
A_0062	Citric acid	Citric acid	311	HMDB0000094	10,207	8,996	9,952	10,096	10,484	9,215	6,600	6,821	6,728	6,325	6,176	6,005
A_0063	*cis*-Aconitic acid	cis-Aconitic acid	643757	HMDB0000072	176	168	190	163	173	145	120	121	122	97	89	83
A_0064	Isocitric acid	Isocitric acid	1198	HMDB0000193	376	336	332	361	347	308	217	231	248	153	132	126
C_0001	Urea	Urea	1176	HMDB0000294	4,478	3,992	4,704	6,307	6,150	5,486	4,044	4,159	4,363	5,326	5,964	5,720
C_0002	Gly	Gly	750	HMDB0000123	21,359	19,353	20,550	32,819	34,344	32,175	30,445	30,538	30,745	39,110	42,541	40,727
C_0003	Putrescine	Putrescine	1045	HMDB0001414	395	351	384	610	634	569	337	355	341	392	447	462
C_0004	Ala	Ala	602	HMDB0000161,HMDB0001310	38,964	35,911	38,766	52,148	56,326	42,819	97,734	97,646	100,871	103,759	105,047	108,151
C_0005	β-Ala	b-Ala	239	HMDB0000056	3,655	3,548	3,739	3,955	4,012	3,584	4,064	4,014	4,171	4,313	4,237	4,623
C_0006	Sarcosine	Sarcosine	1088	HMDB0000271	176	122	147	227	170	219	236	255	396	297	307	337
C_0007	*N*,*N*-Dimethylglycine	DMG	673	HMDB0000092	N.D.	49	50	80	79	81	82	88	82	94	108	116
C_0008	γ-Aminobutyric acid	g-Aminobutyric acid	119	HMDB0000112	1,592	1,488	1,610	1,823	1,781	1,685	796	797	861	925	925	936
C_0009	Choline	Choline	305	HMDB0000097	N.D.	126	212	N.D.	N.D.	N.D.	390	171	200	N.D.	N.D.	N.D.
C_0010	Ser	Ser	617	HMDB0000187,HMDB0003406	1,059	1,212	942	1,516	1,718	1,917	2,731	2,768	2,826	4,190	4,253	4,333
C_0011	Carnosine	Carnosine	439224	HMDB0000033	40	44	N.D.	67	63	60	44	45	42	51	59	45
C_0012	Creatinine	Creatinine	588	HMDB0000562	125	129	135	176	167	165	153	153	137	161	170	186
C_0013	Pro	Pro	614	HMDB0000162,HMDB0003411	30,549	30,656	32,785	41,022	44,635	37,538	65,739	64,597	67,393	75,083	78,452	78,039
C_0014	Val	Val	1182	HMDB0000883	2,673	2,448	2,757	4,858	4,986	4,590	2,332	2,291	2,275	3,241	3,961	3,562
C_0015	Betaine	Betaine	247	HMDB0000043	861	907	869	1,076	1,438	871	3,068	2,983	3,068	4,247	4,693	4,409
C_0016	Thr	Thr	6288	HMDB0000167	6,926	6,604	7,433	11,641	11,800	10,792	9,907	9,843	9,792	11,877	13,178	12,946
C_0017	Homoserine	Homoserine	12647	HMDB0000719	63	61	69	92	128	89	97	112	102	116	127	128
C_0018	Betaine aldehyde	BTL	249	HMDB0001252	N.D.	N.D.	N.D.	N.D.	N.D.	N.D.	N.D.	N.D.	N.D.	N.D.	N.D.	N.D.
C_0019	Cys	Cys	594	HMDB0000574,HMDB0003417	458	561	870	1,529	1,537	875	3,763	3,259	2,592	4,270	4,937	7,344
C_0020	Hydroxyproline	Hydroxyproline	5810	HMDB0000725	32,106	28,953	31,776	44,170	47,256	36,124	68,896	68,313	68,809	80,113	82,188	81,341
C_0021	Creatine	Creatine	586	HMDB0000064	3,546	3,428	3,661	5,840	6,292	5,419	2,777	2,910	3,061	3,727	4,055	4,208
C_0022	Ile	Ile	791	HMDB0000172	12,653	11,346	13,045	20,754	22,112	18,571	19,808	19,000	19,585	23,216	24,812	24,024
C_0023	Leu	Leu	857	HMDB0000687	10,722	9,559	10,711	17,070	18,743	15,736	15,328	15,101	16,434	18,554	20,654	20,096
C_0024	Asn	Asn	236	HMDB0000168,HMDB0033780	59,652	55,364	60,971	90,169	91,715	75,878	101,877	101,439	102,681	117,825	121,034	123,274
C_0025	Ornithine	Ornithine	389	HMDB0000214,HMDB0003374	1,391	1,392	1,537	2,212	2,422	1,786	5,928	5,793	5,862	7,480	7,820	8,018
C_0026	Asp	Asp	424	HMDB0000191,HMDB0006483	25,405	22,226	24,920	32,361	32,415	28,395	7,498	7,814	8,380	5,584	5,556	5,839
C_0027	Homocysteine	Homocysteine	778	HMDB0000742	44	52	45	79	63	50	117	105	96	53	63	83
C_0028	Adenine	Adenine	190	HMDB0000034	21	20	18	28	28	28	20	15	26	21	23	19
C_0029	Hypoxanthine	Hypoxanthine	790	HMDB0000157	N.D.	N.D.	N.D.	N.D.	N.D.	N.D.	N.D.	N.D.	N.D.	N.D.	N.D.	N.D.
C_0030	Spermidine	Spermidine	1102	HMDB0001257	78	73	78	58	72	35	115	119	120	107	104	120
C_0031	Gln	Gln	738	HMDB0000641,HMDB0003423	47,915	40,282	47,693	75,274	69,760	74,365	383	390	469	357	352	362
C_0032	Lys	Lys	866	HMDB0000182,HMDB0003405	500	505	509	1,022	1,012	985	540	550	562	810	991	908
C_0033	Glu	Glu	611	HMDB0000148,HMDB0003339	152,207	147,357	157,118	197,236	195,453	186,064	78,259	80,014	81,865	70,779	69,679	74,376
C_0034	Met	Met	876	HMDB0000696	2,021	1,591	2,118	3,625	3,550	3,271	1,831	1,858	1,676	2,718	2,735	3,437
C_0035	Guanine	Guanine	764	HMDB0000132	N.D.	N.D.	N.D.	N.D.	N.D.	N.D.	N.D.	N.D.	N.D.	N.D.	N.D.	N.D.
C_0036	His	His	773	HMDB0000177	3,233	3,017	3,400	5,163	5,520	4,337	10,094	9,917	9,944	11,909	12,770	13,119
C_0037	Carnitine	Carnitine	85	HMDB0000062	171	166	133	198	205	193	171	181	229	270	302	290
C_0038	Phe	Phe	994	HMDB0000159	1,549	1,408	1,510	2,494	2,516	2,302	1,369	1,320	1,276	1,692	2,166	2,016
C_0039	Arg	Arg	6322	HMDB0000517,HMDB0003416	10,787	10,229	11,249	18,143	19,383	14,945	27,233	26,962	27,067	32,784	35,017	35,164
C_0040	Citrulline	Citrulline	9750	HMDB0000904	662	628	647	906	1,016	762	1,970	1,931	1,963	2,455	2,609	2,657
C_0041	Tyr	Tyr	1153	HMDB0000158	2,892	2,590	2,970	4,779	5,118	4,054	7,050	6,969	7,013	9,004	9,701	9,755
C_0042	*S*-Adenosylhomocysteine	SAH	439155	HMDB0000939	109	105	123	162	171	182	96	126	119	107	133	140
C_0043	Spermine	Spermine	1103	HMDB0001256	60	N.D.	16	N.D.	N.D.	N.D.	58	51	94	45	61	65
C_0044	Trp	Trp	1148	HMDB0000929	409	395	414	706	729	670	505	510	471	719	890	812
C_0045	Cystathionine	Cystathionine	834	HMDB0000099	1,203	1,231	1,096	1,623	1,761	1,588	4,984	5,309	5,197	6,227	6,319	6,831
C_0046	Adenosine	Adenosine	60961	HMDB0000050	202	194	217	271	271	219	186	170	197	188	193	217
C_0047	Inosine	Inosine	6021	HMDB0000195	50	40	42	63	61	57	55	54	53	65	73	72
C_0048	Guanosine	Guanosine	6802	HMDB0000133	61	69	60	91	110	98	112	107	117	153	144	156
C_0049	Argininosuccinic acid	ArgSuccinate	16950	HMDB0000052	600	569	585	1,199	1,220	1,076	547	540	526	692	651	712
C_0050	Glutathione (GSSG)	GSSG	65359	HMDB0003337	2,815	3,151	3,112	5,981	6,520	5,429	2,934	2,840	3,931	8,877	8,020	5,627
C_0051	Glutathione (GSH)	GSH	124886	HMDB0000125	38,131	38,019	36,930	34,181	35,274	32,788	29,436	29,022	27,838	15,666	19,383	27,226
C_0052	*S*-Adenosylmethionine	SAM	34755	HMDB0001185	461	426	452	684	712	679	458	427	458	460	429	460

ID consists of analysis mode and number. 'C' and 'A' showed cation and anion modes, respectively.
N.D. (Not Detected): The target peak or metabolite was below detection limits.
N.A. (Not Available): The calculation was impossible because of insufficience of the data.
